# How context alters value: The brain’s valuation and affective regulation system link price cues to experienced taste pleasantness

**DOI:** 10.1038/s41598-017-08080-0

**Published:** 2017-08-14

**Authors:** Liane Schmidt, Vasilisa Skvortsova, Claus Kullen, Bernd Weber, Hilke Plassmann

**Affiliations:** 1 Sorbonne-Universités-INSEAD Behavioural Lab, 75005 Paris, France; 2 INSERM, U960 Laboratoire de Neuroscience Cognitive, Economic Decision-Making Group, Ecole Normale Supérieure, 75005 Paris, France; 3 Center for Economics and Neuroscience, University of Bonn, 53127 Bonn, Germany; 4 Department of Epileptology, University Hospital Bonn, Bonn, 53127 Germany; 5 INSEAD, Marketing Area, 77300 Fontainebleau, France

## Abstract

Informational cues such as the price of a wine can trigger expectations about its taste quality and thereby modulate the sensory experience on a reported and neural level. Yet it is unclear how the brain translates such expectations into sensory pleasantness. We used a whole-brain multilevel mediation approach with healthy participants who tasted identical wines cued with different prices while their brains were scanned using fMRI. We found that the brain’s valuation system (BVS) in concert with the anterior prefrontal cortex played a key role in implementing the effect of price cues on taste pleasantness ratings. The sensitivity of the BVS to monetary rewards outside the taste domain moderated the strength of these effects. These findings provide novel evidence for the fundamental role that neural pathways linked to motivation and affective regulation play for the effect of informational cues on sensory experiences.

## Introduction

Past research has shown that information from the environment affects our value-related expectations and in turn how we report experiences across a variety of sensory domains, including pain^1^, vision^2^, smell^3^, hearing^4^ and taste^5–7^. Yet it is unknown how the brain translates changes in expected value into changes in experienced value. Our previous work investigated either the effect that an experimental condition such as a change in the price of a wine has on neural activity or how neural activity correlated with reported changes in taste pleasantness ratings^6^. However, localizing the neural pathways that formally explain how price cues can bias taste pleasantness experiences requires the joint consideration of these two univariate effects. This can be done with brain mediation analysis, which is an important approach because it provides fundamental insights into the *mechanisms* underlying cue-based expectancy effects of price information on (taste) pleasantness.

Concepts in decision neuroscience show that the brain’s valuation system (BVS, i.e., the ventromedial prefrontal cortex (vmPFC) and the ventral striatum (vStr))^8–10^ encodes both expected and experienced value^11, 12^ and in turn might be crucial for translating value expectations into experienced value. Furthermore, meta-analyses of a related phenomenon in a different sensory domain — that is, how information about the efficacy of a treatment promotes analgesia — showed that expectations induced by placebo cues led to increased activity in the BVS^1^ and brain regions in the lateral and anterior prefrontal cortex^13^ involved in the regulation of affective states^14, 15^ and stimulus values^16^.

Building on these findings, we hypothesized that value-coding and affective regulation processes are an important antecedent causing expectancy effects of price cues to occur. Specifically, we predicted that: (1) Value-based modulation of taste pleasantness ratings should be formally mediated by activity in the BVS, (2) if this is the case, these effects should be stronger the more sensitive an individual’s BVS is to reward, and (3) the BVS is not the only brain mediator of price-cue effects. Other brain systems linked to affective regulation should also contribute to their occurrence.

**Figure 1:**
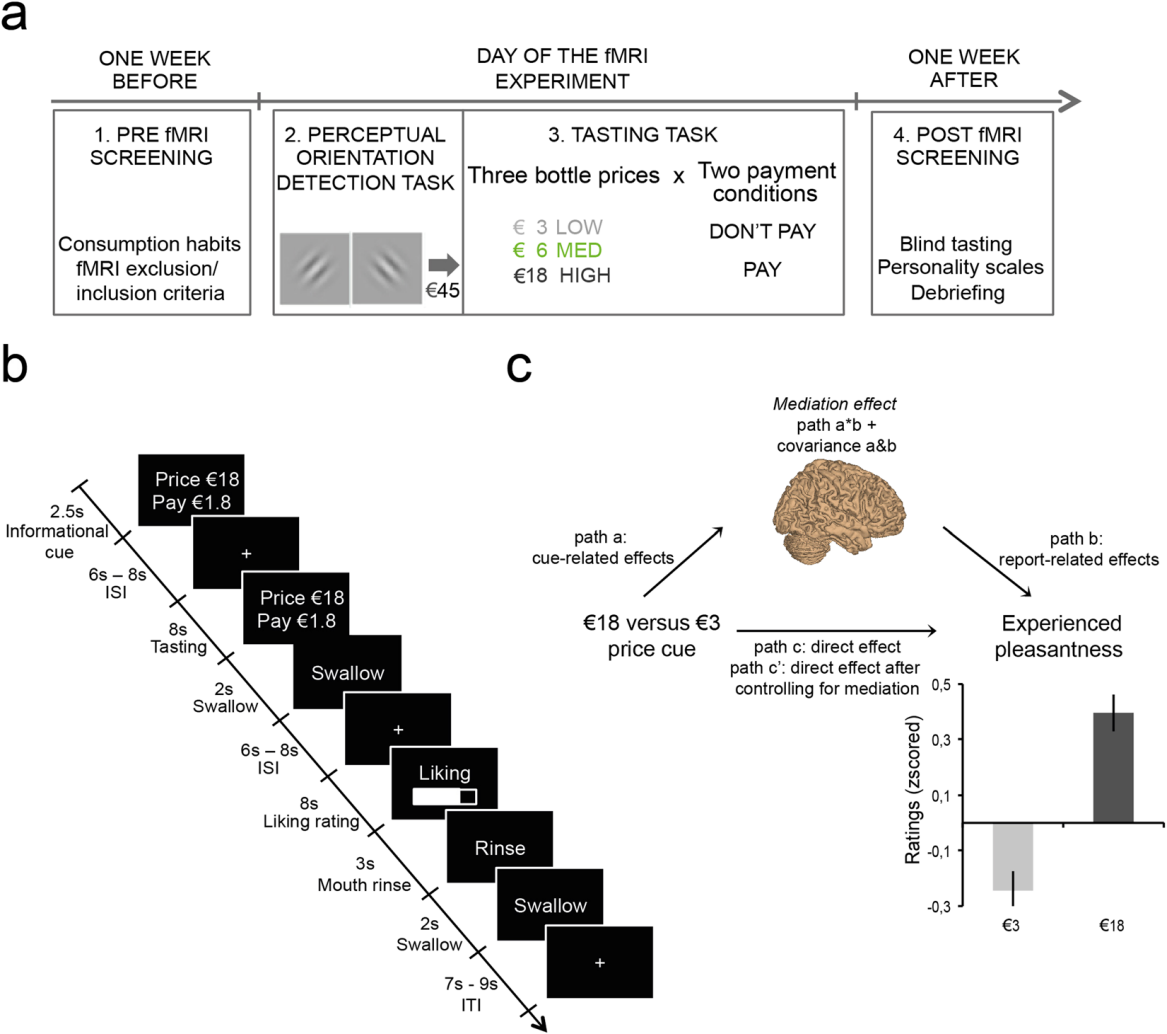
Experimental procedure, wine tasting task and results in N = 30 participants. (**a**) Experimental procedure: The experiment consisted of three events spread over two weeks. (**b**) Wine tasting task: Screenshots depict events within one trial with durations in seconds. Each trial started with the display of the price and payment condition. Following a jittered interstimulus interval (ISI) with a fixation cross on the screen, 1.25 ml of wine was tasted via a tube and subsequently swallowed. Participants rated on a visual analogous scale their experienced pleasantness. They then rinsed their mouth with a water-like neutral liquid. Trials were separated by a jittered inter-trial interval (ITI) during which a fixation cross was displayed on the screen. (**c**) Mediation framework and behavioral results from N = 30 participants: Multilevel whole-brain mediation involved high and low price cue trials (€18, €3) as predictor variables, trial-by-trial beta images of brain activation at time of wine tasting as a mediator variable, and experienced taste pleasantness ratings as a dependent outcome variable depicted in the bar graphs. Error bars correspond to the standard error of the mean (SEM). Note that the effect of price cue was linear across the three price conditions, replicating prior work^6^. There was no effect of payment condition (see SI Figs S1–S3), which was fit into the model as a covariate of non-interest alongside wine type.

To investigate these three hypotheses, participants took part in a previously used wine-tasting task assessing the effects of price cues on experienced taste pleasantness^6^ (Fig. 1a). We conducted a multilevel brain mediation analysis^17, 18^ that provides a formal test of mediation (i.e., experimental manipulation–brain–behaviour links)^18–21^. We further leveraged the fact that some of our participants also took part in a previously used probabilistic monetary decision-making task^22^. The design of this task allowed us to capture responses of the BVS to the receipt of monetary rewards that we could use as a measure of individual differences in BVS sensitivity outside the domain of taste rewards. For these participants, we conducted a multilevel whole-brain moderated mediation analysis^20, 23^ testing whether individual differences of BVS reward sensitivity moderated price cue effects.

## Results

### Price cues increased experienced taste pleasantness ratings

First, we replicated the effect of price cues on experienced pleasantness ratings^6^. As shown in Fig. 1, we found that higher prices induced greater experienced pleasantness for identical wines (linear effect of price cue: β = 0.45, *SE* = 0.03, *p* < 0.001, 95% CI [0.39–0.51], Table 1). Our design also allowed us to explore whether such price expectancy effects depended on whether the participants had to pay for the wines they consumed. There was no significant main effect of payment condition (pay vs. consume for free) (β = −0.03, *SE* = 0.03, *p* = 0.43, 95% CI [−0.09–0.04]) and no significant interaction between price and payment condition (β = −0.01, *SE* = 0.03, *p* = 0.68, 95% CI [−0.07–0.05]) (see Supplementary Fig. S1). Moreover, we compared ratings influenced by price cues to baseline ratings that were sampled in a blind tasting a week after the fMRI session (M_blind_ = 5.03, SEM = 0.23). This comparison revealed that the price cue effects were driven by decreases in experienced pleasantness of low-priced (€3, M_informed_ = 4.19, SEM = 0.20) wines rather than increases in experienced pleasantness of high-priced (€18, M_informed_ = 5.21, SEM = 0.21) wines (paired, two-tailed *t*-test: €3 M_informed-blind_ = −0.83, SEM = 0.28 vs. €18 M_informed-blind_ = 0.18, SEM = 0.30, *t*(29) = −5.2, *p* < 0.001) (see Supplementary paragraph 1.2 and Fig. S2).

**Table 1:** Linear mixed-effects model for experienced pleasantness ratings during fMRI scanning.

Model information	Number of observations	Fixed-effects coefficients	Random-effects coefficients	Covariance parameters	*p*	95% CI	
	3225	12	30	2			
	AIC	BIC	Log likelihood	Deviance			
	12886	122970	−6428.5	12857			
Fixed-effects predictors	ß	SD	t	DF			
						LL	UL
Intercept	4.68	0.18	26.21	3213	<0.001	4.33	5.03
Trial number	−0.17	0.03	−5.22	3213	<0.001	−0.23	−0.11
Wine	−0.08	0.03	−2.75	3213	0.006	−0.15	−0.03
Price cue	0.45	0.03	13.91	3213	<0.001	0.39	0.51
Payment	−0.03	0.03	−0.79	3213	0.43	−0.09	0.04
Trial number by Wine	0.06	0.03	1.83	3213	0.07	−0.00	4 0.12
Trial number by Price cue	−0.05	0.03	−1.43	3213	0.15	−0.11	0.02
Trial number by Payment	−0.05	0.03	−1.51	3213	0.13	−0.11	0.01
Wine by Price cue	−0.04	0.03	−1.42	3213	0.16	−0.11	0.02
Wine by Payment	−0.01	0.03	−0.30	3213	0.76	−0.07	0.05
Price cue by Payment	−0.01	0.03	−0.41	3213	0.68	−0.07	0.05
Trial by Price by Payment	0.03	0.03	0.93	3213	0.35	−0.03	0.09
**Random-effects covariance parameters**		**95% CI**					
**Subject (30 levels)**	**ß**	**LL**	**UL**				
Intercept	0.96	0.74	1.25				
**Error**	**ß**	**LL**	**UL**				
Residual	1.74	1.7	1.78				

### Brain mediators of price cue effects on experienced pleasantness ratings

Second, we investigated which brain areas formally explained the effect of price cues on experienced pleasantness ratings. We conducted a multilevel whole-brain mediation analysis (https://github.com/canlab/MediationToolbox). As outlined in Fig. 1, the mediation analysis jointly considers two types of predictions at the time of wine tasting: (1) how price cues affect brain activity (path a) and (2) how brain activity predicts experienced pleasantness ratings (path b). Multilevel mediation is inferred if the direct effect from price to experienced pleasantness ratings (path c) is significantly reduced (i.e., inferred from a two-tailed bootstrap test) after controlling for the product of path a and path b coefficients within participants in addition to their covariance (cov (a,b)) across participants (path c’)^24^. Note that our main focus is the third path (i.e., the mediator path, a × b+ cov(a,b) = c-c’) and whether our regions of interest are not only involved in this mediating path but also active in the first two paths. Against this background, and for the sake of completeness, we report the results for all three paths (i.e., a, b and a × b+ cov(a,b)) of the regression model.

### Price cue effect on brain activity at time of tasting (i.e., path a regression)

**Figure 2:**
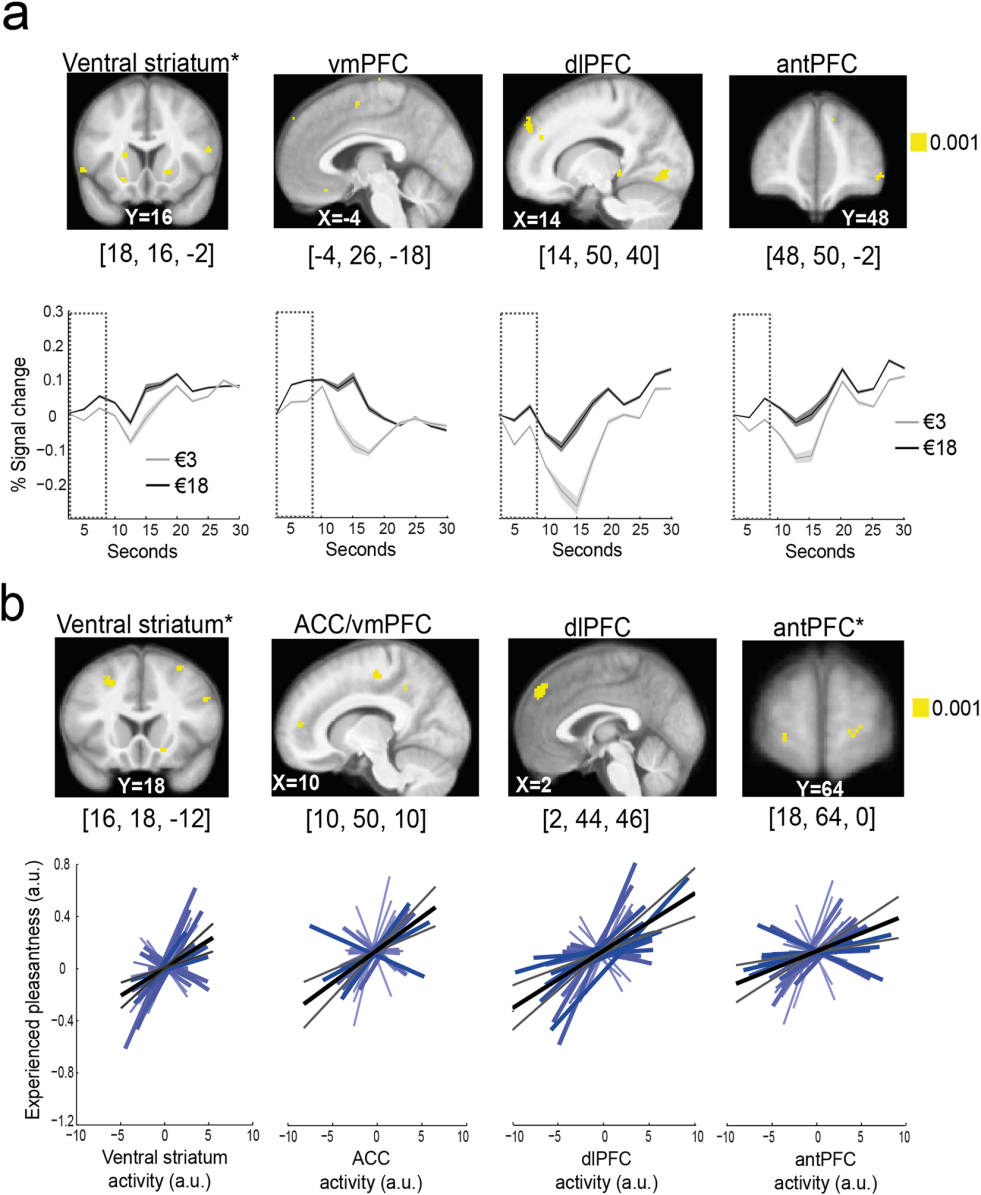
Brain responses to price cue and experienced pleasantness ratings averaged across N = 30 participants (*p_FWE_ < 0.05). Significant voxels are displayed for visualization purposes in yellow at *p* < 0.001 uncorrected, and are superimposed on the average anatomical brain image. The [*x*, *y*, *z*] coordinates correspond to MNI coordinates and are taken at maxima of interest. (**a**) Path a: price cue–related effect on brain activity. The line graphs depict time courses across seconds for activations in the reported [*x*, *y*, *z*] MNI coordinates. The dotted squares denote the 8-second-long wine tasting period. Shaded errors represent confidence intervals (means ± intersubject SEM). (**b**) Path b: brain activity underpinning experienced pleasantness ratings. Line graphs depict individual variations in path b coefficients (blue lines) with the group regression slope (grey line) for the activations in the reported [*x*, *y*, *z*] MNI coordinates. Grey lines represent confidence intervals (95%). ACC/vmPFC: anterior cingulate cortex/ventromedial prefrontal cortex; antPFC: anterior prefrontal cortex; dlPFC: dorsolateral prefrontal cortex.

Path a assessed the price cue–related responses, which correspond to the relationship between price cue and brain activity. This effect is equivalent to the contrast high versus low price cue from standard univariate analyses (see Supplementary paragraph 2.2 and Supplementary Fig. S4). As shown in Fig. 2a, significant activations were found within the brain’s valuation system, including the vmPFC and the bilateral vStr, in line with previous findings^6^ (Table 2). Other regions that displayed strong responses to tasting high- versus low-priced wines included the dorsolateral prefrontal cortex (dlPFC) and the lateral anterior prefrontal cortex (antPFC, BA 10) extending into the medial antPFC at a lower threshold; the primary gustatory cortex (i.e., insula); and semantic (Broca’s and Wernicke’s areas), motor, somatosensory and visual brain regions (precuneus and occipital cortex) (Supplementary Table S9).

**Table 2:** Path a-, b- and axb-related brain activations.

Region	BA	Size	*x*	*y*	*z*	*Z*
**Path a activation**						
Ventral striatum		17	18	16	−2	8.10
**Path b activation**						
Anterior PFC	10	20	−22	62	−8	8.48
Ventral striatum		9	16	18	−12	8.08
**Path ab + cov** (**a**,**b**) **activation**						
Ventral striatum		7	14	8	−16	7.87
Anterior PFC	10	9	28	64	−6	7.83
vmPFC		37	−4	38	−16	7.73

*Note*: The table was obtained using whole-brain multilevel mediation analysis at onset of wine tasting in N = 30 subjects. Regions listed survived a whole-brain threshold of *p* < 0.001 uncorrected at the voxel level and small volume correction of p_FWE_ < 0.05. BA: Brodmann area; vmPFC: ventromedial prefrontal cortex; PFC: prefrontal cortex.

### Brain activity predicting experienced taste pleasantness at time of wine tasting (i.e., path b regression)

Next, we looked for brain responses that predicted experienced taste pleasantness ratings. Importantly, this path b regression analysis controlled for the effect of price cue on experienced taste pleasantness ratings. Thus, path b assessed brain activity that underpins endogenous variations in experienced taste pleasantness during wine tasting. We found that activity in the anterior cingulate cortex (ACC) adjoining the vmPFC, the right vStr (Table 2) extending into the nucleus accumbens (NAcc) and the hippocampus correlated significantly with variations in experienced taste pleasantness ratings irrespective of the price cue effects on these ratings (Fig. 2b). Additional activation patterns were found in the lateral and medial part of the dorsal prefrontal cortex, and in more central regions in the bilateral anterior PFC (BA 10), somatosensory (posterior insula), middle temporal lobe, visual and motor cortex regions (Supplementary Table S10).

### Brain mediators of price cue effects on experienced pleasantness (i.e., path a × b + cov(a,b) regression)

**Figure 3:**
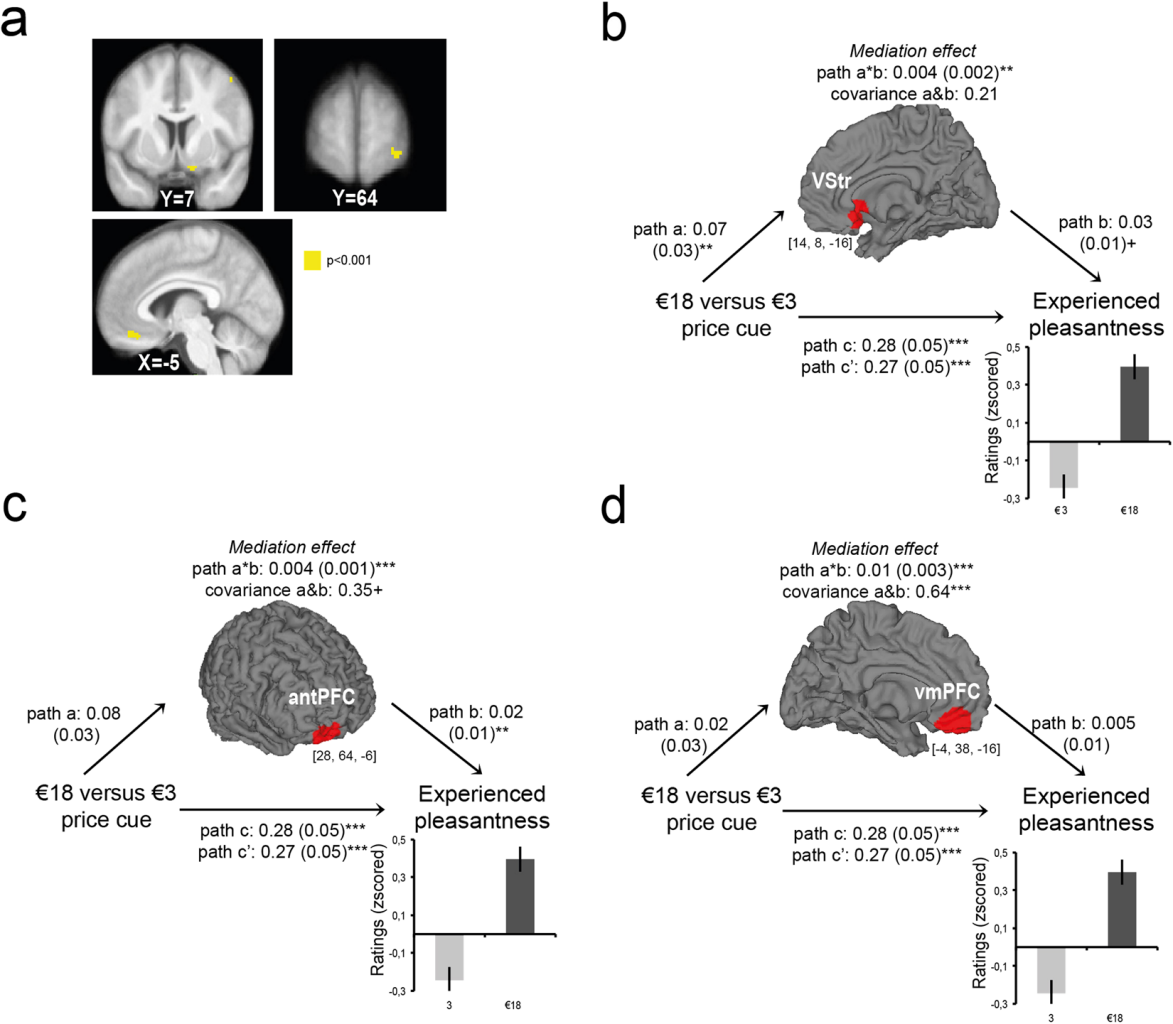
Brain mediators and moderators of price cue effects in N = 30 participants. (**a**) BOLD activity in the ventral striatum, anterior prefrontal cortex and ventromedial prefrontal cortex mediated price cue effects on experienced pleasantness ratings (*p*_FWE_ < 0.05). Significant voxels are displayed for visualization purposes in yellow at *p* < 0.001 uncorrected, and are superimposed on the average anatomical brain image. (**b**–**d**) Multilevel mediation path diagram across N = 30 participants for the three brain mediators of price cue effects: (**b**) the ventral striatum, (**c**) the ventromedial prefrontal cortex (vmPFC) and (**d**) the anterior prefrontal cortex (antPFC). The [*x*, *y*, *z*] coordinates correspond to Montreal Neurological Institute (MNI) coordinates and are taken at maxima of interest. Average path coefficients (a × b (SEM)) and the correlation of a&b coefficients (cov) across participants denote the joint activation in paths a and b at ***p < 0.001, **p < 0.01 or ^+^ *p* < 0.05 two-tailed. Note that multilevel mediation effects can be driven either by significant path a and b co-activation or by covariance of path a and b coefficients.

Third and most important, we looked for brain regions that formally mediated the relationship between price cue and experienced taste pleasantness ratings. We found significant activations in the vmPFC, the right vStr and the anterior PFC (BA10) (Fig. 3, Table 2, Supplementary Table S11) that satisfied the three criteria for formal mediation as outlined above^24^. Importantly, the regression coefficients for path a and path b were significantly correlated (*r* = 0.64, *p* < 0.01, two-tailed) for the vmPFC cluster, suggesting that its mediating role was driven by covariance. This is a common observation using multilevel whole-brain mediation analyses^19^. It implies that the vmPFC voxels consistently explained the effect of price cues on experienced pleasantness on the population level, although individual path a and path b coefficients varied in strength.

Note that the path c’ regression assessing the direct effect of price cue on experienced pleasantness remained significant after controlling for activations of the brain mediators, suggesting a partial mediation role. Other brain mediators that were activated only by path a × b + cov(a,b) included temporal cortex, insula, and motor and visual brain regions (Supplementary Table S11).

### General, task-independent sensitivity of the BVS moderates brain mediators of price cue effects on experienced pleasantness

**Figure 4:**
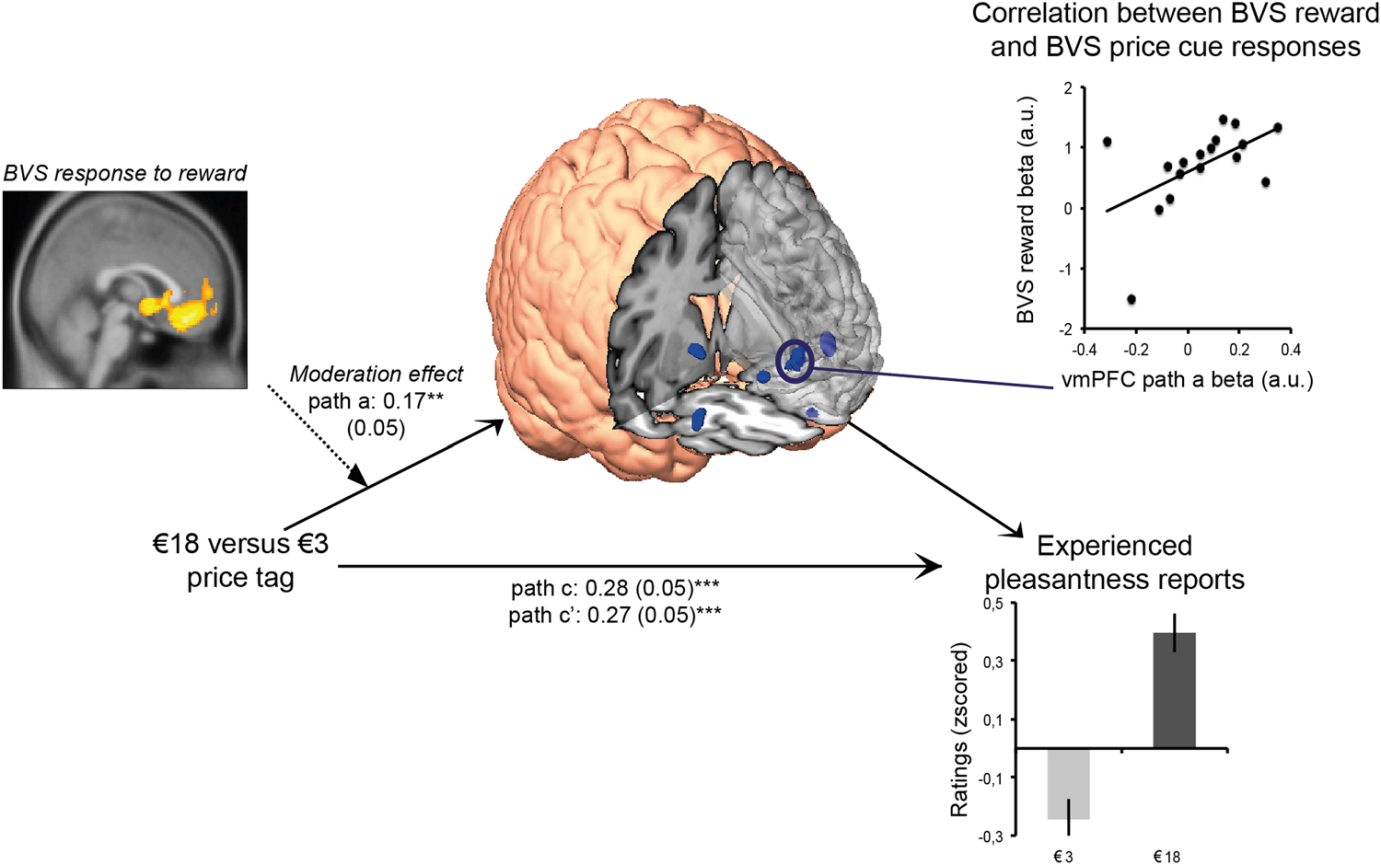
Whole-brain moderation of the price cue–related effect (path a) during wine tasting in a subset of N = 17 participants. The yellow and orange voxels depict significant brain activation by experienced reward during an independent monetary decision-making task, and are displayed at a threshold of *p*_FWE_ < 0.05 corrected for multiple comparisons on the cluster level. Blue voxels depict brain regions of the BVS that were positively moderated by the reward-related activation of the BVS during the monetary decision-making task. They are displayed at a threshold of *p* < 0.001 uncorrected at the voxel level for visualization purposes. The path a coefficient corresponds (SEM) to the moderated price cue effect on the vmPFC activation (**p < 0.01, two-tailed), highlighted by a dark blue circle. All voxels are superimposed on the average anatomical brain image. The scatterplot shows the correlation between the vmPFC path a response (*p*_FWE_ < 0.05) and the reward-related BVS response.

To provide further evidence for the key role that the BVS plays in implementing price cue effects on experienced taste pleasantness, we investigated the role of individual differences in sensitivity of the BVS as assessed by its neural response to the receipt of monetary rewards in a different task. In line with our hypothesis, we found that neural activation of the BVS positively moderated price cue effects in the ventromedial prefrontal cortex and ventral striatum (Fig. 4). Other path a–related brain activations moderated by individual sensitivity of the BVS involved the anterior prefrontal cortex, amygdala, dorsomedial prefrontal cortex, insula, periaqueductal grey and middle temporal gyrus (Supplementary Table S12).

## Discussion

The goal of this paper was to show that activity in the BVS (i.e., the vStr and vmPFC) plays a key role for price cue effects on experienced pleasantness ratings. We used a state-of-the-art methodological approach — multilevel, moderated mediation analysis — providing novel evidence that (1) activity in the BVS formally mediated price cue effects and (2) individual differences in BVS sensitivity assessed in an independent monetary decision-making task moderated such price cue effects. Taken together, these two findings imply that the BVS plays a general key role in such expectancy effects.

An interesting question is whether both regions of the BVS play the same role in price cue effects on experienced pleasantness. In several meta-analyses^8–10^ the vStr and vmPFC consistently and jointly correlated with the size of values assigned to different stimuli. However, the results from decision neuroscience also suggest that these two brain regions have distinct roles in value coding. The vmPFC is suggested as a neural substrate for the representation of an overall goal value of options for choice — that is, the magnitude and probability of an expected pleasurable experience^25^. In contrast, it has been suggested that the ventral striatum functions as a common motivational node in the brain representing the (1) desirability of reward, which could be interpreted as a “wanting” of the reward for choice^26, 27^, and (2) deviations between expected and experienced reward (i.e., the so-called prediction error)^28^.

In addition, the pain placebo literature (which is related to our question because it investigates the impact of informational cues from the environment on pain experiences) also suggests that two regions might play distinct roles: In their review Wager and Atlas proposed that the vmPFC might be an important hub to integrate all incoming information into “a coherent schema that informs and is informed by responses at other processing levels”^1^. This notion is in line with the idea that the vmPFC integrates different information into a valuation signal^11^. Further, Wager and Atlas’s review also suggests that the vStr might be specifically linked to motivational processes during valuation — that is, a “wanting” to believe that one has received a painkiller does indeed translate into a less painful experience. This idea is in line with the linking of striatal activation to dopamine functioning that has been shown to be important for valuation^29^ and for placebo effects^30, 31^. Further research is needed to better understand the distinct roles that the vStr and the vmPFC play for cue-based expectancy effects and the contributions of motivation and dopamine to such effects.

Our findings that the anterior PFC formally mediated price cue effects and that another brain region — the dlPFC — consistently activated in path a and path b regressions provide first evidence for the recruitment of neural pathways involved in cognitive control of affective states for the occurrence of cue-based expectancy effects. Functional magnetic resonance imaging (fMRI) studies from the field of social and affective neuroscience have provided evidence that the anterior PFC and dlPFC are part of a set of frontal cortex regions that underpin the cognitive regulation of affective states^14, 15, 32, 33^ and are associated with a variety of executive functions such as working memory^34, 35^ and cognitive control in the sense of goal-directed action selection^36, 37^.

Interestingly, the anterior PFC is considered a “gateway” region that integrates incoming information from the environment with individual information from long-term memory^35^, supporting the idea that this system may implement expectancy effects of cues from the environment, such as the price of a taste sample or the white lab coat of the experimenter administering a placebo drug. One possibility could be that participants might recruit this brain region when reflecting on the external information from the price cue and their subjective beliefs and memories about how expensive and less expensive wines should taste. Additional evidence favouring this idea comes from a growing body of research on pain suggesting that frontal cortex brain regions involved in cognitive regulation processes overlap with brain regions associated with placebo analgesia^13^. For example, the anterior PFC and dlPFC are activated under the administration of a placebo drug in concert with verbal suggestion^33, 38–41^. Interestingly, the anterior PFC price cue mediator region indeed overlaps with a similarly located brain region activated under placebo effects of a sham anxiolytic drug^33^. Yet the similarity of brain activation across different experimental contexts and tasks does not allow generalization of inferences about the specific underlying psychological processes^42^. We call for future studies that further test the causal contributions of cognitive processes such as emotion regulation and cognitive control for expectancy effects.

An interesting and novel finding of our study was that our three regions of interest (the vmPFC, vStr, and anterior PFC) implement price cue effects in the taste domain most prominently early in the tasting period (i.e., when modelling the taste period within a boxcar of 3 seconds). Extending this boxcar model to the full 8 seconds of the tasting period shifted our activation patterns in the mOFC part of the vmPFC to more dorsal parts of the vmPFC and attenuated the involvement of the vStr (see SI paragraph 2.4 for details). Notably, the most prominent brain mediator of price cue effects was found in the dorsal anterior cingulate cortex, a region most prominently and broadly involved in top-down attentional control^43^ (see Supplemental Fig. S9).

We would like to raise one important limitation of our study. We have reported results in our a priori and independently defined regions of interest using family-wise error corrections (in the main text) and have reported all our analyses for path a, path b, and mediating axb path activations for the whole-brain mediation analysis at a threshold of *p* < 0.001 uncorrected (in the SI). However, none of the regions survived more conservative whole-brain cluster-level FWE corrections. It is important to note, though, that our main results referring to brain mediators are based on an analysis that differs from standard univariate analyses because it jointly considers the path a and path b regressions. In other words, for each path regression (e.g., path a: the contrast of high vs. low price) we control for activation associated with the remaining path regressions (e.g., path b: the parametric effect of pleasantness ratings), rendering the analysis more conservative for false positives compared to standard univariate analyses that consider these effects separately.

Finally, in an exploratory analysis (see SI paragraph 2.6 for details), we compared whether the mediators of price cue effects on experienced taste pleasantness in the BVS and anterior PFC were located within a set of brain regions reported by a recent meta-analysis to activate under placebo analgesia^20^. We found first preliminary evidence for an overlap between the systems implementing expectancy effects in the pain and taste pleasantness domains. Thus, our exploratory findings may suggest that brain mechanisms implementing placebo effects might share common neural pathways across sensory domains. If further research directly comparing placebo effects across domains could confirm these exploratory findings, it might help to reconcile previous debates contrasting whether there are variations in placebo responses^13^ or whether they are similar^1, 44^. Our preliminary findings give rise to the idea that there might be differences in how the brain reacts to different contextual cues from the environment (i.e., the path a and path c responses), but the neural pathways that translate those cues into experiences (i.e., that are mediating them) might be shared. We call for future research that investigates in more detail this idea of shared neural pathways that causally implement expectancy effects across opposite sensory domains.

In conclusion, our brain mediation analysis shed light on the neural substrate that underlies and moderates how an informational cue (i.e., the price) acts as a placebo by triggering a change in a person’s sensory experience, keeping the actual experience constant. This approach parallels placebo research done on pain and extends knowledge about the mechanisms of placebo effects to the appetitive domain, present in everyday life behaviours and perceptions. By conducting a mediation analysis we went beyond standard univariate approaches of previous work done on the effects of price cues on taste pleasantness ratings. This allowed us to disentangle the neural and behavioral placebo responses from the neural mechanisms implementing them. Thus, our findings contribute to a better general understanding of mind–brain–body interactions, and how they shape human behaviour in everyday life.

## Material and Methods

The study was approved by Bonn University’s Institutional Review Board. All experiments were performed in accordance with the standards of the Declaration of Helsinki. Participants gave written and informed consent before enrolling in the experiment.

### Participants

We recruited 54 healthy participants (21 male, 33 female; mean age = 29.1 years, SE = 1.1 years) via public advertisement at Bonn University. The sample size was chosen to be similar to those generally employed in the field. Participants were paid a show-up fee of €35 and received additional remuneration based on their performance in the study. Participants were screened for liking and at least occasionally drinking red wine. Standard fMRI inclusion criteria were applied to select participants. These included right-handedness, normal to corrected-to-normal vision, no history of substance abuse or any neurological or psychiatric disorder, and no medication or metallic devices that could interfere with performance of fMRI (Supplementary Tables S13 and S14 for additional participant information).

Twenty-four participants were excluded before the data was analysed due to the following predefined exclusion criteria: head movement (≥3 mm; N = 17), incomplete wine tasting task data (N = 4) and insufficient orbitofrontal cortex coverage (N = 3), which was a priori defined as one region of interest based on previous findings and a meta-analysis on the brain’s valuation system^6, 8^.

Therefore, the analyses we used to investigate brain mediators of price cue effects on experienced taste pleasantness are based on a total of N = 30 participants (15 male, 15 female; mean age = 29.6 years, SE = 1.6 years). Note that in a previous study of our group^6^ we had a lower exclusion rate due to head movement because head movement was tracked with a coil around the larynx of each participant, which made the instruction not to move extremely salient.

In a follow-up analysis, we explored the role of individual differences in the sensitivity of the BVS, as sampled in an independent monetary decision-making task. To construct this individual difference measure (described in more detail below), we leveraged the fact that 17 of the 30 participants were also scanned with fMRI while performing a previously used monetary decision-making task^22^. Thus, the respective analyses were based on the subset of participants who took part in both tasks (i.e., 9 male, 8 female; mean age = 28.4 years, SE = 2.6 years).

### Procedure

The experiment consisted of four events spread over two weeks (Fig. 1a). Participants were recruited via flyer advertisement for an fMRI experiment investigating how participants would evaluate wines under different conditions. The experimental procedure consisted of the following steps.

### Screening via phone interview (1 week before fMRI scanning)

During the phone interviews participants were screened for common fMRI exclusion criteria. We also screened out wine experts and participants who did not like to taste wines or had dietary restrictions preventing them from doing so.

### Perceptual orientation detection task (day of fMRI experiment before fMRI session)

Prior to scanning, participants performed a version of the Gabor orientation discrimination task previously used to test participants’ attention and perceptual learning skills^45^. The task duration was 20 minutes and was performed outside the fMRI scanner. The goal of this task was to implement a seemingly performance-based task that allowed us to have participants earn “house money” for the main tasting task, in which they were asked to spend some of the money they earned. In this Gabor orientation task participants were instructed to compare two Gabor patches and to decide whether they had the same or a different orientation without receiving feedback on the correctness of their responses. We used this task because it allowed us to adapt the task difficulty to each individual’s performance by varying the tilt. This was important because it allowed us to keep the performance and thus the performance-based payment constant for each participant. In other words, the task difficulty was adjusted such that the performance of each participant was around 60% to 65% correct answers. Every participant earned a total of €45 for his or her performance in the Gabor orientation task. The money was given to each participant after the task as a mix of coins and €5 bills in a bowl. The experimenter told the participants that the bowl would be kept in the fMRI control room and that money would be taken out each time the participant needed to spend money in the subsequent task.

### Wine tasting task

The wine tasting task was our main paradigm to investigate price cue effects on experienced taste pleasantness coding (Fig. 1). Participants repeatedly sampled 1.25 ml of three wines with the same retail price (€12). We had two experimental conditions: (1) We manipulated the bottle price information given to participants in each trial (i.e., €3, €6, €18), and (2) we manipulated whether participants could sample the wines for free or whether they had to pay a price that was 10% of the indicated bottle price (i.e., €0.3, €0.6, or €1.8). Importantly, each of the three wines was assigned to all experimental conditions over the course of the experiment, and we did not predict differences of our manipulations between the wines (i.e., wine was not treated as an experimental factor but was controlled for in all analyses reported below).

Taken together, we applied a 3 (bottle price €3, €6, €18) × 2 (pay/no pay) within-participant experimental design. Each experimental condition was repeated 18 times (i.e., six times for each of the wines), resulting in a total of 108 trials. These trials were split up into three fMRI sessions of 36 trials with a duration of 30 minutes each. Importantly, within each fMRI session the order of the six experimental conditions (3 price × 2 payment conditions) and the three wine types was pseudo-random: The same wine was never tasted across successive trials, and repetitions of price and payment conditions were restricted to a maximum of two successive trials.

In total, participants consumed 135 ml of wine (less than a glass), and the total duration of the fMRI session was about 1.5 hours. Structural scans were acquired at the end of the fMRI session for 10 minutes. Screenshots of events within one trial are displayed in Fig. 1. Each trial started with the display of the price and payment condition information onset (2.5 s). A jittered inter-trial interval (ITI) (6 to 8 s) separated the price and payment information onset from the tasting period. During tasting the information about price and payment condition was again displayed on the screen, and the wine was delivered via an in-house–built electronic syringe pump system. The syringes filled with the respective liquids were placed on a MR-compatible system directly in the bore of the scanner to make the feeding tubes as short as possible. They were connected via a hydraulic tube system to electronic syringes in the control room. During this period, participants were instructed to swirl the liquid in their mouths (for a period of 8 s) and evaluate its pleasantness. They were also instructed to swallow only when the word “swallow” was displayed on the screen (2 s), to reduce head movement due to swallowing response as much as possible. After an ITI (6 to 8 s) participants were asked to enter their ratings of the pleasantness of the wine sample on a nine-point Likert scale from unpleasant to pleasant (8 s). After the rating, they rinsed their mouths with a neutral water-like liquid with a taste similar to saliva (containing 1 g/l potassium chloride + 1 g/l sodium bicarbonate + distilled water) (3 s) and swallowed (2 s). Subsequent trials were separated by a jittered ITI (7 to 9 s) during which a fixation cross was displayed on the screen. Participants saw the information via goggles and indicated their responses using a response box system (both NNL, Bergen, Norway). Bonn University’s in-house presentation software was used as experimental software to present the events and record responses.

### Blind tasting task (one week after the fMRI session)

One week after the fMRI session, participants performed a blind tasting evaluating pleasantness and taste of the three wines used during the fMRI session, without price or payment condition information present. They tasted 5 ml of each wine and rated pleasantness (one question: How much do you like this wine? not at all to a lot) and taste (two questions: How would you describe its taste? ordinary to extraordinary; inferior to superior) using a nine-point Likert scale. Participants were paid €10 for their participation in the blind tasting session.

### Monetary decision-making task

We used a monetary decision-making task that had effectively elicited activations in the brain’s valuation system in the past^22^ (see Supplementary paragraph 2.3 and Fig. S6). In this task, participants were instructed that their goal was to win as much money as possible by finding a circle in one out of a varying number of boxes displayed on the screen. Importantly, this task was non-hypothetical, and participants received additional payoffs based on their performance. That is, when participants guessed the right box as containing the circle, they were rewarded with a payoff of 10 cents; otherwise they won nothing. That means that each guess was associated with different known reward probabilities as a function of the number of boxes on the screen. In other words, the reward probability was 100% if only one box was shown, 50% for two boxes, 33% for three boxes, and 25% for four boxes. Because the reward probabilities were known, no learning was involved in this task. In total, participants earned €5 for performing the task, and they made on average €4.61 ( ± €0.16) based on the number of correct guesses.

### Behavioral data analysis

All statistical tests were conducted with the Matlab Statistical Toolbox (Matlab 2015a, MathWorks). A linear mixed-effects model (using the *fitlme* function in Matlab) was fit for experienced pleasantness ratings with fixed effects for trial number (coded 1 to 36 per wine to correct for possible effects over number of tastings for each wine), wine (coded 1, 2 or 3 to test for differences in liking linked to the type of wine irrespective of price cue and payment condition), price (coded 1, 2 or 3), payment condition (coded 0 for free and 1 for pay), all possible two-way interactions, one three-way interaction trial by price by paying, and uncorrelated random effects for intercept grouped by subject (coded 1 to 30). All regressors were z-scored across trials for each subject. Results are reported in Table 1. A similar analysis was conducted for reaction times, and the results are reported in Supplementary Table S1.

### Image acquisition

T2*-weighted echo planar images (EPI) with BOLD contrast were acquired on a 1.5T Siemens Magnetom Avanto scanner. We further applied a special sequence designed to optimize functional sensitivity in the orbitofrontal cortex (OFC). This consisted of a tilted acquisition in an oblique orientation at 30° to the AC-PC line. In addition, we used an eight-channel phased array coil that yields a significant signal increase in the OFC over the standard head coil. To cover the whole brain with a repetition time of 2.5 seconds, we used the following sequence parameters: 31 axial slices; 3-mm slice thickness; 0.3-mm inter-slice gap corresponding to 10% of voxel size. T1-weighted structural images were also acquired, co-registered with the mean EPI image, segmented and normalized to a standard T1 template, and averaged across all participants to allow group-level anatomical localization. The first three volumes of each session were discarded to allow for T1 equilibrium effects. Preprocessing consisted of spatial realignment, normalization using the same transformation as structural images, spatial smoothing using a Gaussian kernel with full width at half maximum of 8 mm, and high-pass temporal filtering (filter width 128 s).

### fMRI Analysis

fMRI data was analysed using the Statistical Parametric Mapping software (SPM12; Wellcome Department of Imaging Neuroscience). Multilevel mediation analysis was conducted using the Mediation Toolbox (https://github.com/canlab/CanlabScripts). We performed two analyses. In the first analysis, we were interested in identifying the formal brain mediators of price cue effects on experienced pleasantness ratings. In the second analysis, we were interested in whether the brain mediators from the first analysis were moderated by each participant’s BVS sensitivity, as measured by the neural response to monetary reward receipt during an independent monetary decision-making task.

### What were the brain mediators of price cues on experienced taste pleasantness ratings?

The analysis involved the following two steps.

### Single-trial fMRI analysis

For whole-brain mediations we used a single-trial or “single-epoch” analysis approach^46, 47^. Building on the implementation of the single-trial analysis for whole-brain multilevel mediations of cue effects on pain perception^20, 23^, we applied this approach to the modelling of price effects on experienced pleasantness. A general linear model (GLM) design matrix estimated the magnitude of single-trial responses at the time of wine tasting (boxcar durations: 3 seconds) with separate regressors for each trial. Note that we have chosen a time window of 3 seconds rather than the full tasting period of 8 seconds for two reasons: (1) The literature suggests that cognitive modulation of flavour responses happens quite fast (i.e., in the first 2 to 4 seconds)^3, 6, 48^ and (2) the computations in the brain underlying value coding in our main regions of interest, the BVS, were also shown to occur early during the value-coding time, as compared to other, more cognitive regions involved later during the value-coding time^16, 49^.

Regressors of non-interest included “dummy” regressors coding for the intercept of each run, and the linear drift across time within each run. To correct for movement artefacts, a total of 24 head movement regressors were included in the model as additional nuisance regressors of non-interest. They counted six estimated head movement parameters from image realignment (x, y, z, roll, pitch, and yaw), their mean-centred squares, and their derivatives and squared derivatives. Previous work featuring univariate^50^ and multivariate analyses^23^ of fMRI data have applied this approach and showed that it contributes to reducing noise variance, violations of normality and autocorrelation^51^.

Boxcar functions for each trial were convolved with the canonical hemodynamic response function. Note that estimations obtained by single-trial analyses are susceptible to noise from acquisition artefacts (sudden motion, scanner pulse artefacts, etc.).

In line with previous studies using the single-trial approach for whole-brain multilevel mediations, variance inflation factors (VIFs) were calculated to estimate the design-induced uncertainty due to colinearity with nuisance regressors^20, 23^. The VIF quantifies how much the variance of single-trial regression coefficients is inflated due to covariance with the nuisance covariates. For each regressor *k* (i.e., wine tasting onset) in the single-trial model, the VIF is calculated based on VIF_k_ = 1/1 − *R*_*k*_^2^, with *R* ^2^ corresponding to the determination coefficient of each *k* against the nuisance regressors. On average we excluded 1.06 trials (SD = 2.8) per participant (VIF ≥ 2.5). The VIFs of included trials ranged from 1.005 to 2.05. Single-trial beta images were then used as mediator variable *M* for the mediation analyses.

### Multilevel whole-brain mediation analysis

Mediation analysis extends standard univariate fMRI analyses by jointly considering (1) the brain activation in response to price cues and (2) brain activation predicting experienced taste pleasantness ratings. This is achieved by including a mediator variable *M* corresponding in the current study to brain activation at time of wine tasting. Under the null hypothesis of no mediation, the two joint effects—price cue effect on brain activation and brain activation on experienced pleasantness ratings—are uncorrelated. A mediator variable is suggested to formally explain the covariance between a predictor variable *x* (i.e., price cue) on an outcome variable *y* (i.e., experienced pleasantness rating). Taken together mediation analysis jointly tests three effects expressed by the following regression equations:

1 $${\rm{path}}\,{\rm{c}}:y=cx+{e}_{y}$$

2 $${\rm{path}}\,{\rm{a}}:m=ax+{e}_{m}$$

3 $${\rm{path}}\,{\rm{b}}:y=bm+c^{\prime} x+e^{\prime} y$$

The variables *x*, *y* and *m* correspond to trial-by-trial data vectors containing each subject’s experienced pleasantness ratings (*y*), price information (*x*) and data from each voxel at time of tasting (*m*). The variables e_y_, e_m_ and e’_y_ denote residual variance for each of the three regression analyses, respectively. We considered only the contrasts between the two extreme price cue conditions for the predictor variable *x* (e.g., €18 vs. €3). This was motivated by two facts: (1) that the goal of this paper was to understand the brain mediators of price cue effects and (2) that this contrast generated the largest effect on the behavioural outcome variable *y* (i.e., experienced taste pleasantness ratings). However, to test the robustness of our findings, we also conducted an analysis using all three price levels as the *x* variable. This analysis yielded results similar to those of our categorical analysis (see SI Section 2.3 for details).

The first regression, called path c, corresponds to the direct (or total) effect of the experimental manipulation (*x* = price information) on behaviour (*y* = experienced taste pleasantness ratings). The second regression, called path a, tests the relationship of the experimental manipulation (*x* = price information) on brain activity (*m* = activity in single-trial beta images). This effect is equivalent to the contrast high versus low price cue from standard univariate GLM analyses. The third regression, called path b, assesses the relationship between brain activity at time of tasting (*m*) and behaviour (*y* = experienced pleasantness ratings), controlling for the experimental manipulation (*x* = price information). This effect, because it jointly controls for the effect of the experimental manipulation (i.e., price information), is also called report-related response identifying the brain regions that predict endogenously driven variations in experienced pleasantness. To control for additional experimental manipulations induced by wine type and payment conditions, these two variables were included in the mediation model as covariates of no interest.

Single-level mediation is assessed by the product of path a and path b coefficients: a × b = c − c’, with c’ corresponding to the direct effect of price tag on experienced pleasantness, controlling for the brain activity at time of wine tasting (the mediator variable).

Importantly, the current study used a multilevel mediation analysis, which accounts for both within- and between-participant variations by treating participant as a random effect. This involves testing on a first level — across trials within each participant — the dynamic variations between experimental manipulation, behaviour, and brain activity, and on a second level for consistency of these variations across participants, allowing for population inferences. Crucially, multilevel mediation is inferred by the product of path a and path b coefficients and an additional component corresponding to the covariance of path a and path b estimates across participants (e.g., participants with strong path a effects also show strong path b effects) as expressed by equation 9 in Kenny *et al*.^19^:

4 $${\rm{mean}}({\rm{a}}\times {\rm{b}})={\rm{mean}}({\rm{a}})\,\times \,{\rm{mean}}({\rm{b}})+{\rm{cov}}({\rm{a}},{\rm{b}}).$$

Thus, a multilevel mediation driven by covariance can identify voxels that consistently explain the effect of price cues on experienced taste pleasantness on the group level, although the path a and path b coefficients are heterogeneous on the individual level (e.g., negative for some and positive for others)^19, 20, 23^. We performed bootstrapping to test the significance of the mediation effects^52, 53^. This involved estimating the distribution of individual path coefficients by randomly sampling with replacement 10,000 observations from the matrix of [a, b, c, c’, a × b] path coefficients. Two-tailed *p*-values were calculated from the bootstrap confidence intervals. Bootstrapping is a non-parametric test for mediation analyses, because path coefficients are not normally distributed^20, 23^.

In line with common thresholds for whole-brain mediation analysis, we considered whole-brain activations of continuous voxels at *p* < 0.001 uncorrected with a cluster extend threshold of 5 voxels^20, 23^. Path-related brain activations were further corrected for multiple comparisons by calculating family-wise error (FWE)-corrected *p*-values (p_FWE_ < 0.05) within 10-mm-radius spheres centred around the Montreal Neurological Institute (MNI) space coordinates of a priori defined regions of interest (ROIs) located in the BVS and anterior prefrontal cortex. Specifically, the vmPFC ROI was defined by the MNI coordinates [*x* = −2, *y* = 48, *z* = −16], and the ventral striatum ROI was defined by the MNI coordinates [*x* = 12, *y* = 10, *z* = −16]. Both coordinates are reported by a meta analysis for subjective value-related brain activation^8^. The anterior prefrontal cortex ROI was defined by the MNI coordinates [x = 28, y = 50, z = −8] reported previously for placebo effects of sham anxiolytic drugs on affective regulation^33^.

### Do brain mediators of price cues on experienced taste pleasantness ratings vary as a function of individual differences in the brain’s valuation system?

To test the moderating role of an individual’s neural sensitivity to experienced value, we extracted average beta estimates from 10-mm-radius spheres that were centred on the maxima of activation located in the ventromedial prefrontal cortex (vmPFC), the ventral striatum and the adjacent anterior cingulate cortex (ACC), which activated significantly in response to the receipt of a monetary reward (i.e., experienced value) during the monetary decision-making task (*p*_FWE_ < 0.05, family-wise error whole-brain correction) (Supplementary Table S6). The average of these beta estimates was used as a second-level moderator regressed to path a–related brain responses. We conducted the moderated, multilevel whole-brain mediation analysis in a subset of N = 17 participants for which this data was available. In addition, to check whether individual outliers biased moderations, we extracted the beta estimates for the path a response of the vmPFC (i.e., MNI = [6, 56, 14], SVC *p*_FWE_ < 0.05) and plotted them against the average beta estimates from the BVS moderator ROI described above (Fig. 4).

### Time courses

For all reported time course analyses, we extracted time courses from activations at maxima of interest. The response time courses were estimated using a flexible-basis set of finite impulse responses, separated by one TR of 2.5 seconds.

### Code availability

All statistical tests were conducted with the Matlab Statistical Toolbox (Matlab 2015a, MathWorks). fMRI data was analysed using the Statistical Parametric Mapping software (SPM12, Functional Imaging Laboratory, the Wellcome Trust Centre for NeuroImaging in the Institute of Neurology at University College London). Multilevel mediation analysis was conducted using the Mediation Toolbox (https://github.com/canlab/MediationToolbox).

## Electronic supplementary material

Supplementary information
