# Supplementary material for: How context alters value: The brain’s valuation and affective regulation system link price cues to experienced taste pleasantness
Source: Sci Rep. 2017 Aug 14;7:8098. doi: 10.1038/s41598-017-08080-0 (PMC5556089; doi:10.1038/s41598-017-08080-0)
Supplement: Supplementary file 1 — Supplementary information [file 41598_2017_8080_MOESM1_ESM.pdf]

Supplementary Information

TITLE

How context alters value: The brain's valuation and affective regulation system link price cues to experienced taste pleasantness

Authors and affiliations

Liane Schmidt[*,1,2], Vasilisa Skvortsova[2], Claus Kullen[3], Bernd Weber[3,4,#] and Hilke Plassmann[2,5#]

[1]Sorbonne-Universités-INSEAD Behavioural Lab, 75005 Paris, France
[2]INSERM, U960 Laboratoire de Neuroscience Cognitive, Economic Decision-Making Group, Ecole Normale Supérieure, 75005 Paris, France
[3]Center for Economics and Neuroscience, University of Bonn, 53127 Bonn, Germany
[4]Department of Epileptology, University Hospital Bonn, Germany
[5]INSEAD, Marketing Area, 77305 Fontainebleau, France
[#]joint senior authorship

[*]Correspondence should be addressed to Liane Schmidt, INSERM U960, LNC DEC, ENS, 29, rue d'Ulm, 75005 Paris, France. Email: liane.schmidt@insead.edu

1. Supplemental behavioural analyses and results
   1.1. Experienced taste pleasantness ratings during fMRI scanning
   1.2. Experienced taste pleasantness ratings during blind wine tasting
2. Supplementary fMRI analyses and results
   2.1. Taste-responsive brain regions (contrast: wine > water-like control)
   2.2. Price cue–responsive brain regions during wine tasting (contrast: €18 > €3)
   2.3. Whole-brain mediation analysis for the linear price cue effect on experienced taste pleasantness ratings
   2.4. Whole-brain mediation analysis across the full 8-second wine tasting period
   2.5. Experienced value–related brain responses during the monetary decision-making task
   2.6. Localizing brain mediators or price cue effects on experienced taste pleasantness within brain regions of interest activated under placebo analgesia
3. Additional tables S8 – S20

1. Supplementary behavioural analyses and results

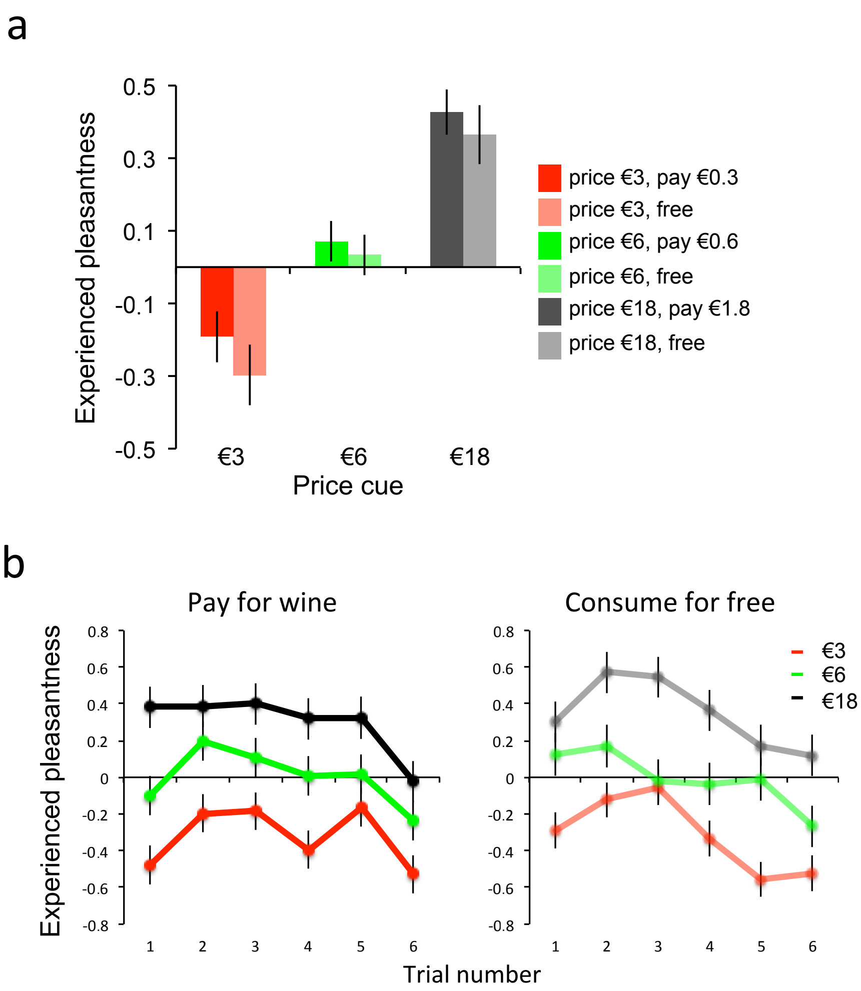

Supplementary Figure S1. Behavioural results from wine tasting task.
(a) Bar graphs depict average experienced pleasantness ratings (z-scored across wine types) for each price and payment (pay/free) condition. Error bars correspond to the standard error of the mean. **(b)** Line graphs depict average experienced pleasantness ratings (z-scored) across the 6 trials of each price condition (€3, €6, €18) for trials during which participants needed to pay for the sample (left graph) and trials during which samples were free (right graph). Error bars correspond to within-subject SEM.

1.1. Experienced taste pleasantness ratings during fMRI scanning

In an additional analysis, we examined whether income predicts the effect of price cue on experienced taste pleasantness ratings. To get at this question we correlated the difference in experienced taste pleasantness ratings of wine cued with a high price (€18) versus a low price (€3) and income information given by 23 participants (coded 1 to 7 for < €15,000 to > €100,000). Seven participants were excluded because they declined to respond to the income questionnaire. Permutation tests revealed no significant correlations between income and the effect of price cue on experienced taste pleasantness (r = –0.10, p = 0.63, 95% CI [–0.36–0.34]).

1.2. Experienced taste pleasantness ratings during blind wine tastings

One week after fMRI scanning, participants underwent a blind tasting evaluating the taste pleasantness of the three wines used during fMRI scanning without price information. Participants tasted 5 ml of each wine and rated their experienced pleasantness (one question: How much do you like this wine? not at all to a lot) and taste pleasantness (two questions: How would you describe its taste? ordinary to extraordinary; inferior to superior) using a nine-point Likert scale. Participants were paid €10 for their participation in this session.

These blind ratings were used as a baseline measure for experienced taste pleasantness without price cues present. This measure allowed us to investigate whether price cue effects were driven by decreases of pleasantness ratings due to low price cues or increases due to high price cues. A linear mixed-effects model was fit for differences in experienced pleasantness ratings between informed (i.e., price cued during fMRI session) and blind (i.e., post-scanning session) wine tastings, with fixed effects for price, payment condition, wine type, price by payment condition, price by wine type, payment condition by wine type and uncorrelated random effect for the intercept grouped by subjects (Table S1). Post hoc one-sampled paired *t*-tests further assessed differences in experienced taste pleasantness reported in each condition of the fMRI experiment (€3, €6, €18 * pay, no pay) versus experienced taste pleasantness during the blind wine tastings, respectively.

As displayed in Supplementary Table S1 and Supplementary Figure S2, we found a significant main effect of price cue (β = 0.63, p = 0.01; 95% CI [0.12–1.12]), indicating that experienced taste pleasantness ratings decreased relative to baseline (i.e., experienced pleasantness reported during blind tastings) as price cues decreased. Post hoc paired *t*-tests confirmed that the difference (i.e., experienced pleasantness of informed tasting – blind tasting) was driven by the low price cues (price €3: $t(29) = –2.94$, $p = 0.006$; price €6: $t(29) = –1.4$, $p = 0.18$; price €18: $t(29) = 0.63$, $p = 0.52$) compared to high price cues (€3 vs. €18: $t(29) = –5.2$, $p < 0.001$).

Supplementary Table S1: Linear mixed-effects model for differences in experienced pleasantness ratings under the influence of price cues (i.e., during fMRI scanning) versus in a blind tasting

Model information	Number of Observations	Fixed effects coefficients	Random effects coefficients	Covariance parameters			
	540	7	30	2			
	AIC	BIC	Log Likelihood	Deviance			
	2215.2	2253.8	−1098.6	2197.2			
						95% CI	
Fixed effects predictors	β	SD	*t*	DF	*p*	LL	UL
Intercept	−1.40	0.63	−2.18	533	.03	−2.64	−0.14
Wine	0.005	0.26	0.02	533	.98	−0.49	0.51
Payment condition	−0.13	0.53	−0.24	533	.80	−1.18	0.92
Price cue	0.63	0.26	2.43	533	.01	0.12	1.12
Wine by Payment condition	0.08	0.18	0.47	533	.63	−0.27	0.44
Wine by Price tag	−0.05	0.11	−0.46	533	.64	−0.27	0.16
Payment condition by Price tag	−0.03	0.18	−0.17	533	.86	−0.38	0.32
Random effects covariance parameters		95% CI					
Subject (30 Levels)	β	LL	UL				
Intercept	1.4034	1.0667	1.8462				
Error	β	LL	UL				
Residual	1.7236	1.621	1.8326				

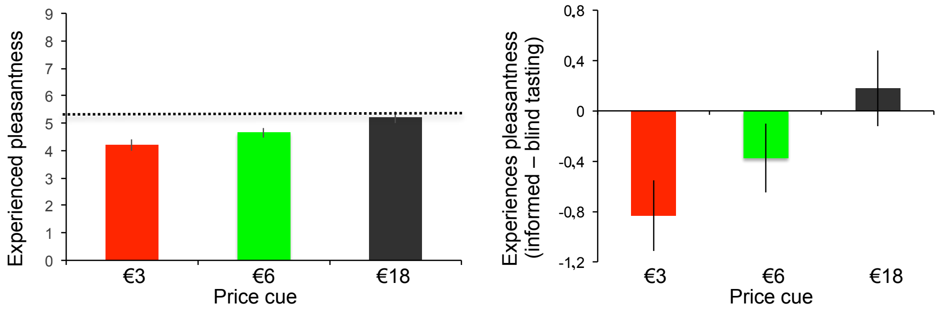

Supplementary Figure S2: Experienced taste pleasantness ratings during fMRI scanning and relative to blind wine tastings. Bar graphs on the left depict average experienced pleasantness ratings for the three price (€3, €6 and €18) conditions of the fMRI experiment. The dotted line indicates experienced pleasantness of each wine during the blind wine tastings. The bar graphs on the right depict the differences in experienced pleasantness (informed – blind wine tasting) for each price condition. Error bars correspond to SEM.

Further exploration of the results revealed that participants preferred wine 1 (Wine 1 M_{blind} = 5.5, SEM = 0.3) and wine 3 (Wine 3 M_{blind} = 5.4, SEM = 0.4) over wine 2 (Wine 2 M_{blind} = 4.2, SEM = 0.3; wine 1 > wine 2: $t(29) = 2.68$, $p < .05$; wine 3 > wine 2: $t(29) = 2.86$, $p < .01$, two-tailed, paired t-test), with no differences between wine 1 and wine 3 ($t(29) = 0.23$, $p = .82$, two-tailed, paired t-test). Importantly, all wines were paired equally often with all three price levels, and as shown in Supplementary Figure S3, the difference in wine liking did not bias the price cue effect on experienced taste pleasantness during fMRI scanning, which was present in all three wines.

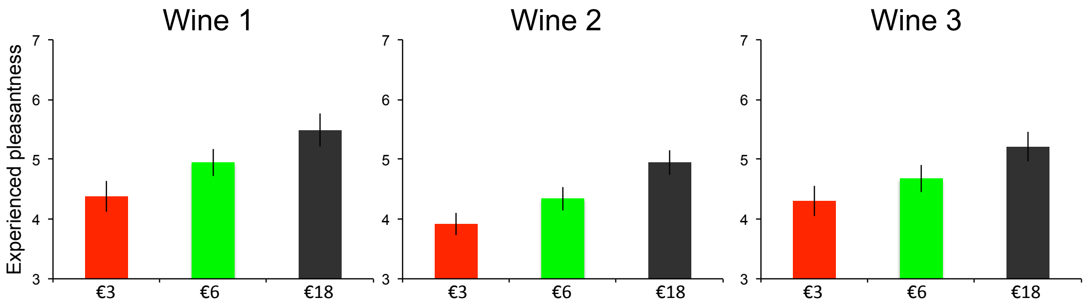

Supplementary Figure S3: Experienced taste pleasantness ratings during fMRI scanning for each wine. Bar graphs depict average experienced pleasantness ratings for the three price (€3, €6 and €18) conditions and wines during the fMRI experiment. Error bars correspond to SEM.

2. Supplementary fMRI analyses and results

2.1. Taste-responsive brain regions (contrast: wine > water-like control liquid)

As a sanity check, in order to localize brain regions that selectively activated in response to the taste of wine, correcting for effects of price, payment condition and wine type we used a general linear model (GLM) that assessed brain responses at time of wine tasting versus mouth rinse. Accordingly, GLM1 involved the following seven regressors: onset cue display (boxcar duration 2.5 seconds) parametrically modulated by pay/no-pay condition and price cue; onset ITI (duration 6–8 seconds) parametrically modulated by pay/no-pay condition and price cue; onset wine tasting (duration 3 seconds) parametrically modulated by pay/no-pay condition, price cue and wine type; onset swallow wine (duration 2 seconds); onset experienced pleasantness

rating (duration reaction time); onset rinse mouth with water-like neutral liquid (duration 3 seconds); and onset swallow (duration 2 seconds). Delta functions of onset regressors and parametric modulators were convolved with the canonical hemodynamic response function and regressed against each subject's fMRI data. Linear contrasts were fit into a second-level random-effects analysis, which used paired *t*-tests to identify brain regions that activated more strongly to the taste of wine than to the water-like neutral liquid (contrast: onset wine tasting > rinse with water-like neutral liquid). Findings are displayed in Supplementary Figure S3 and Table S2.

We found significant activations of the nucleus accumbens and the ventromedial/orbitofrontal cortex involving Brodmann areas 25 and 11, which activated more strongly in response to tasting wine than a water-like neutral liquid. At a more lenient threshold of $p < 0.005$ uncorrected ($k = 50$ voxels), activations extended to the dorsolateral and dorsomedial prefrontal cortex, areas of the motor cortex (M1, SMA, premotor cortex) and parts of the precuneus and occipital cortex.

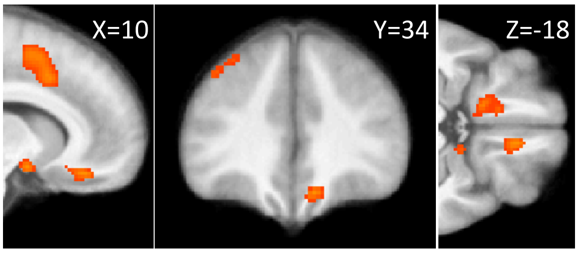

Supplementary Figure S3: Brain regions responding to the taste of wine compared to a water-like neutral liquid. Statistical parametric maps (SPMs) survive a threshold of $p < 0.001$ uncorrected extend threshold $k = 98$, corresponding to $p_{FWE} < 0.05$ on the cluster level. They are displayed at $p < 0.005$, uncorrected at the voxel level ($k = 50$ voxels) superimposed on the average anatomical brain image. MNI (*x*, *y*, *z*) coordinates are taken at maxima of interest located in the ventromedial prefrontal cortex.

Supplementary Table S2: Taste-responsive whole-brain activations (wine > water-like control liquid)

Region	BA	Size	*x*	*y*	*z*	*Z*
vmPFC/OFC	25	98	–12	20	–14	4.10
Premotor cortex		1348	58	8	38	4.89
M1	4	1561	–50	–14	56	5.66
Occipital lobe	19	1822	–24	–96	18	6.15
	18	2910	2	–78	2	6.00

Note: The table was obtained using the contrast between the onset of wine tasting versus onset of mouth rinse with a water-like control liquid. All regions listed survived a whole-brain threshold of $p < 0.001$ extent threshold $k = 98$ corresponding to cluster level $p_{FWE} < 0.05$ (family-wise error). OFC: orbitofrontal cortex; vmPFC: ventromedial prefrontal cortex ; M1: primary motor cortex.

2.2. Price cue–responsive brain regions during wine tasting (univariate contrast: €18 > €3). As another sanity check, we used two additional GLMs to localize brain responses that varied as a function of price cue. To this aim GLM2a was designed in order to match the mediation path a regression. The design matrix consisted of a categorical model with the following onset regressors modelling each event within one trial: cue display (duration 2.5 sec), ITI (duration 6–8 sec), wine tasting (duration 3 sec), swallow wine (duration 0 sec), experienced pleasantness rating (0 sec), rinse (0 sec) and swallow (0 sec). Onsets cue, tasting and swallow wine were further broken down into €3, €6 and €18 price conditions. GLM2b applied the same regressors, but the wine tasting onset was modelled across the whole tasting duration (8 seconds),

and similar boxcar functions were used for the wine swallow (2 seconds), the experienced pleasantness rating (response times), and the rinse (3 seconds) and swallow (2 seconds) onsets. Taken together both GLMs contained a total of 13 onset regressors convolved with the canonical hemodynamic response function and regressed against each subject's fMRI data.

Linear contrasts for each regressor were fit into a second-level random-effects analysis, which used paired *t*-tests to identify brain activity in responses to high-priced (€18) versus low-priced (€3) wines. One-sampled *t*-tests additionally estimated brain responses at onsets cue display, wine tasting and swallow for each price category (€3, €6, €18), respectively. We restricted our search to value-encoding brain regions using a region of interest (ROI) mask that included brain regions reported by an independent study to respond positively to stimulus value[1]. Moreover, we applied small-volume correction using a more restricted region of interest that consisted of a 10-mm-radius sphere centred around the [$x = -4$, $y = 33$, $z = -13$] MNI coordinates, reported by Plassmann et al. (2008)[2], for the contrast €18 versus €3 at time of tasting with a lenient initial whole-brain threshold of $p < 0.05$ uncorrected.

As depicted in Supplementary Figure S4, we found stronger brain activations in response to the tasting of a high-priced wine compared to a low-priced wine (i.e., €18 > €3) in the ventral striatum and the ventromedial prefrontal cortex for GLM 2a (3-second wine tasting) (SVC $p_{\mathrm{FWE}} < 0.05$). These activations were observed during the first 3 seconds of wine tasting. GLM2b revealed that across the full 8 seconds of wine tasting value-related activations shifted to a more dorsal part of the vmPFC (Supplementary Figure S5), with a prominent cluster located in the anterior cingulate cortex at $p < 0.001$ uncorrected.

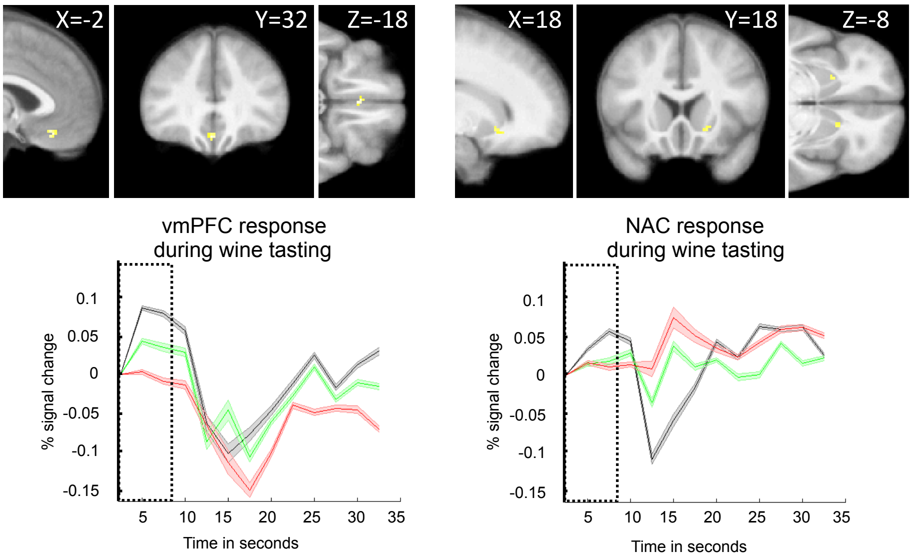

Supplementary Figure S4: Univariate analysis of price cue effects on brain activations at time of wine tasting across the first 3 seconds of the tasting period. Significant voxels for the contrast high versus low price (€18 > €3) are for visualization purposes displayed in yellow at a threshold of $p < 0.001$ uncorrected, masked by an ROI mask comprising brain regions reported by Bartra et al. (2003) to respond positively to stimulus value. SPMs are superimposed on the average T1 anatomical scan. The [*x*, *y*, *z*] coordinates correspond to Montreal Neurological Institute (MNI) coordinates and are taken at maxima of interest. Line graphs depict time courses across seconds of activation in the ventromedial prefrontal cortex (vmPFC) and ventral striatum peak, respectively. Black dotted lines denote the 8-second wine tasting period. Shaded errors represent confidence intervals (means ± intersubject SEM).

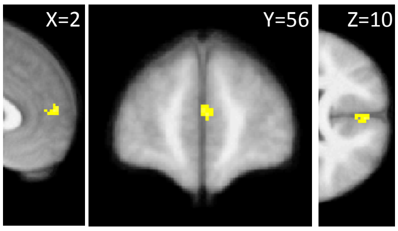

Supplementary Figure S5: Univariate analysis of price cue effects on brain activations at time of wine tasting across the full 8-second tasting period. Significant voxels for the contrast high versus low price (€18 > €3) are displayed in yellow at a threshold of $p < 0.001$ uncorrected. SPMs are superimposed on the average T1 anatomical scan. The [*x*, *y*, *z*] coordinates correspond to Montreal Neurological Institute (MNI) coordinates and are taken at maxima of interest.

2.3. Whole-brain mediation analysis for the linear price cue effect using all three price levels on experienced taste pleasantness ratings

Third, we checked if the results hold for a whole-brain mediation model across all three price conditions for variable *x*. To this end we conducted an analogous analysis as described in the methods section of the main text. The results hold both for whole-brain and ROI-based mediation analyses, and are reported in Supplementary Figure S6 and Supplementary Table S3 below.

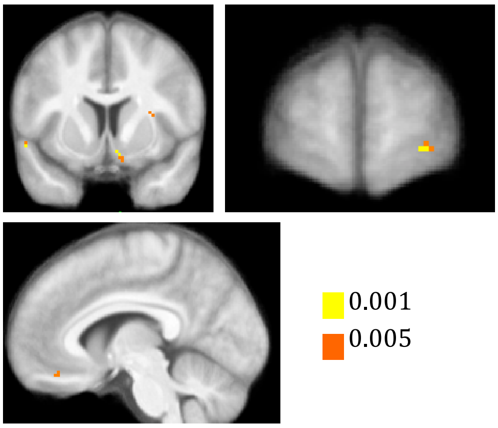

Supplementary Figure S6: Brain mediators and moderators of the linear price cue effect in N = 30 participants. BOLD activity in the ventral striatum, anterior prefrontal cortex and ventromedial prefrontal cortex mediated linear price cue effects on experienced pleasantness ratings ($p_{\mathrm{FDR}} < 0.05$). Significant voxels are displayed for visualization purposes in yellow ($p < 0.001$ uncorrected) and orange ($p < 0.005$ uncorrected), and are superimposed on the average anatomical brain image.

Supplementary Table S3: Path coefficients for mediation analysis of the linear price cue effect

	Path a (X –> M)			Path b (M –> Y)			Path a x b (X –> M –> Y)			Corr(a,b)
	**β**	**SE**	**P**	**β**	**SE**	**P**	**β**	**SE**	**P**	**R**
vmPFC										
[–6 38 –18]	0.01	0.04	0.88	0.01	0.02	0.49	0.0021	0.0007	0.004	0.50
Vstr										
[6 6 –12]	– 0.02	0.04	0.48	–0.01	0.01	0.52	0.0032	0.001	0.00012	0.61
antPFC										
[28 62 –6]	0.05	0.03	0.11	0.02	0.01	0.03	0.0004	0.002	0.0007	0.25

For the sake of completeness, we also conducted two additional mediation analyses for the intermediate and high price conditions (€18 versus €6) and low and intermediate price conditions (€6 versus €3). There was no significant evidence that

the three ROIs play a mediating role at $p < 0.001$ uncorrected and SVC $p_{FWE} < 0.05$. This absence of effects between the two intermediary price levels (i.e., €18 versus €6 and €6 versus €3) can be explained by taking a closer look at the strength of the behavioural effects on these levels (Supplementary Table S4).

Supplementary Table S4: Effect sizes of price cues on experienced taste pleasantness ratings

**Price cue**	**Mean (M)**	**Standard deviation (STD)**	
**€3**	4.17	1.15	
**€6**	4.65	1.01	
**€18**	5.21	1.20	
	**M1 — M2**	**STD_{pooled} = (STD1 ^ 2 + STD2 ^ 2) / 2**	**Cohen's d = (M1 — M2) / STD_{pooled}**
**€6 > €3**	0.48	1.09	0.44
**€18 > €6**	0.56	1.11	0.50
**€18 > €3**	1.04	1.18	0.89

These results imply that the ability of the €6 price tag to serve as a quality signal is much noisier than the two extreme prices. Some participants perceived the €6 wine as expensive and some as cheap. To further strengthen this point, we investigated in more depth the consumption behaviour of our participants. To understand what price levels they perceived as acceptable and would buy most frequently, we asked in the debriefing session a question about how much they spend on average for a bottle of wine. Supporting our point, we found that on average participants spend €6.50, with a standard error of the mean of €0.40 (see also SI Table S8 below). Looking at the distributions we indeed observed that there is a lot of heterogeneity about whether €6 is an acceptable bottle price for our participants (supplementary Fig. S7).

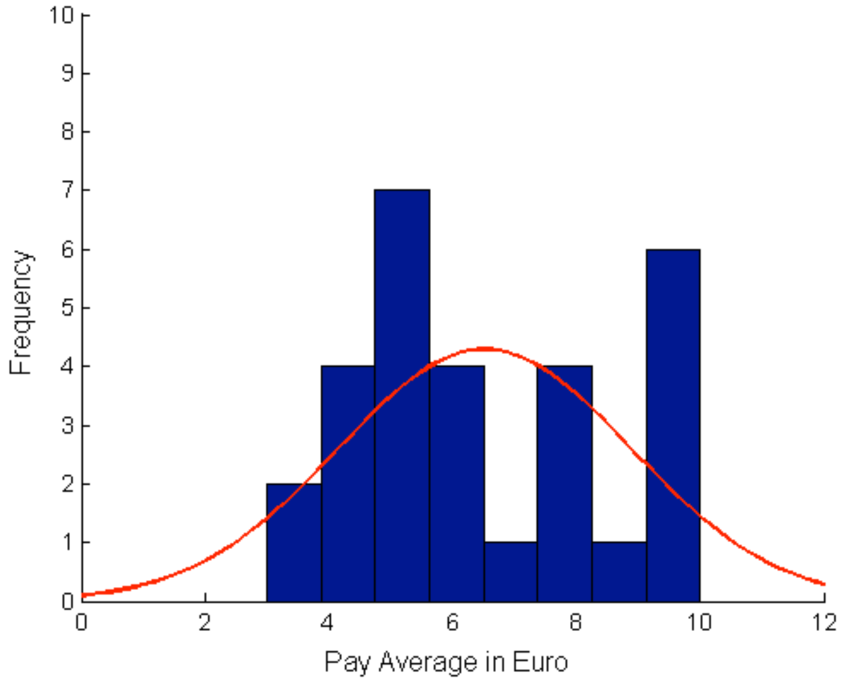

Supplementary Figure S7. Distribution of willingness to pay for a bottle of wine.
Bar graphs show the distribution of participants across different prices they would be willing to pay for a bottle of wine ("What is the price for a bottle of ordinary wine you would purchase?").

Moreover, we also observed that participants, who would spent **less** than €6 for a bottle of wine (N=13), displayed a greater difference in taste pleasantness during the wine tasting task between a €6 and €18 cued wine ($t(12)=3.5$, $p=0.004$), with a borderline significant difference between the €3 and €6 cued wines ($t(12)=2.2$, $p=0.051$). On the contrary, participants, who would spent **more** than €6 for a bottle of wine (N=12), displayed a greater difference between the €3 and the €6 cued wine ($t(11)=2.7$, $p=0.02$) with no difference between the €18 and €6 wines $t(11)=1.8$, $p=0.10$, two-tailed, paired t-tests, see Supplementary Figure S8).

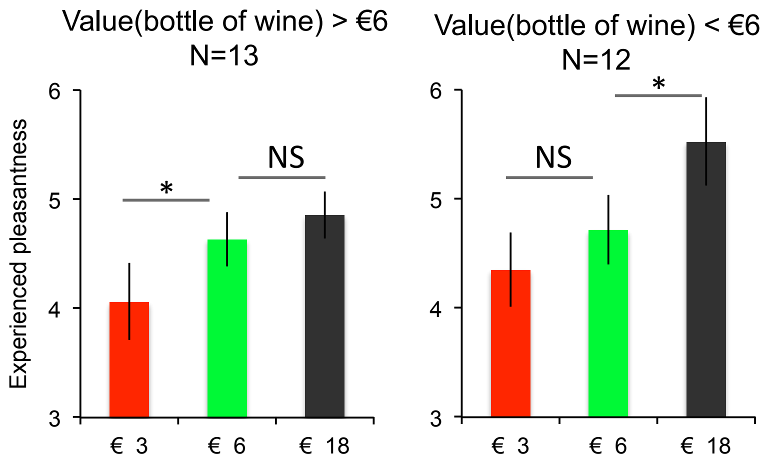

Supplementary Figure S8. Experienced taste pleasantness ratings during fMRI scanning as a function of willingness to pay for a bottle of wine (i.e., value(bottle of wine)). Bar graphs depict average experienced pleasantness ratings for the three price (€3, €6 and €18) conditions during the fMRI experiment. Error bars correspond to SEM. * p<0.05 two-tailed, paired t-test.

Taken together, these findings show that €6 was seen as an ambiguous quality signal for our participant pool. Because the price placebo effects described here depend crucially on the price quality intuition of participants, it makes sense that we could not find evidence for price placebo effects on the brain level between the two intermediary price levels (i.e., €18 versus €6 and €6 versus €3).

2.4. Whole-brain mediation analysis across the 8-second tasting period

Paralleling the univariate analysis of price cue responses reported in paragraph 2.2, we also conducted a whole-brain mediation analysis over the full 8-second wine tasting period. Analysis details are analogous to the main analysis described in the methods section of the main text. We considered whole-brain activations of continuous voxels at $p < 0.001$ uncorrected with a cluster extend threshold of $k = 5$ voxels, and also performed small volume corrections at $p_{\mathrm{FWE}} < 0.05$ using the same ROIs described in the methods section.

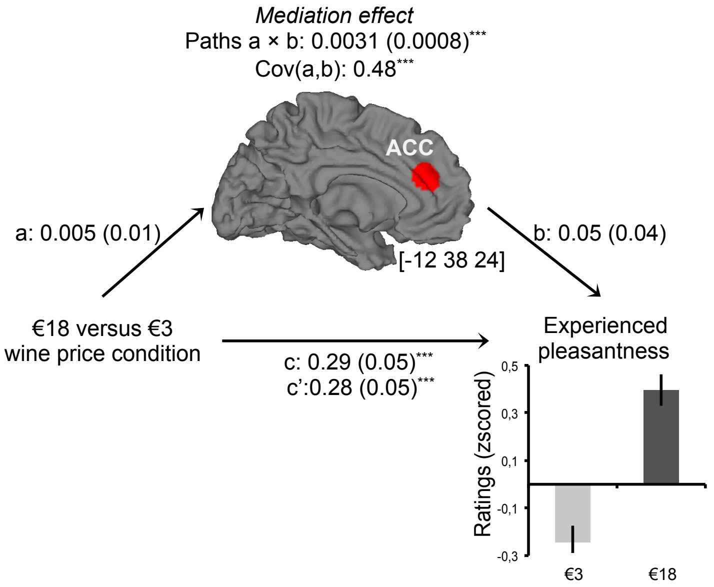

Supplementary Figure S9. Whole-brain mediation of price cue effects across the whole 8 seconds of wine tasting. The path diagram depicts path coefficients for the anterior cingulate cortex (ACC) across N = 30 participants. The [*x*, *y*, *z*] coordinates correspond to Montreal Neurological Institute (MNI) coordinates and are taken at maxima of interest. Average path coefficients (a x b (SEM)) and the correlation of a and b coefficients (cov) across participants denote the joint activation in paths a and b at $^{***}p < 0.001$, $^{**}p < 0.01$ or $^{+}p < 0.05$ two-tailed. Note that multilevel mediation effects can be driven either by significant path a and path b co-activation or by covariance of path a and path b coefficients.

Supplementary Table S5: Mediation path a x b-related brain activation

Region	BA	Size	x	y	z	Z
Postcentral gyrus	1	10	28	-30	76	7.95
Cingulate gyrus		9	18	-8	46	8.30
Cerebrum		6	-28	-68	28	7.07
		5	22	-86	-4	8.24
Precentral gyrus	6	6	-50	-4	32	7.66
Insula	13	5	42	2	-8	7.67
antPFC	10	4	-8	70	14	7.56
MFG/ACC	9	3	-12	38	24	7.17

Note: The table was obtained using a whole-brain multilevel mediation analysis at the time of wine tasting for the full 8-second wine tasting period. All regions listed survived a whole-brain threshold of $p < 0.001$ uncorrected. antPFC: anterior prefrontal cortex; MFG: medial frontal gyrus; ACC: anterior cingulate cortex.

2.5. Experienced value–related brain responses during the monetary decision-making task

To investigate experienced value–related brain responses, a GLM was fitted to brain responses at time of reward feedback. The GLM included the following regressors: two onset regressors at time of choice and reward feedback; parametric modulators involved at time of reward feedback: expected value (expressed as the probability of reward conditioned on the number of boxes to choose from in each trial) and experienced value (expressed as €0/€10 reward dummy coded 0 and 1); and at time of choice onset: hand used to respond (dummy coded 1 and –1 to control for motor responses). Linear contrasts for each regressor were fit into a second-level random-effects analysis, which used one-sampled *t*-tests to identify brain activity for each regressor compared to a baseline.

As shown in Supplementary Figure S10, we found significant activations located in the bilateral ventral striatum and the ventromedial prefrontal cortex extending into the anterior cingulate cortex for experienced value at time of reward feedback ($p_{FWE} < 0.05$, cluster level). Additional significant activations are reported in Supplementary Table S6.

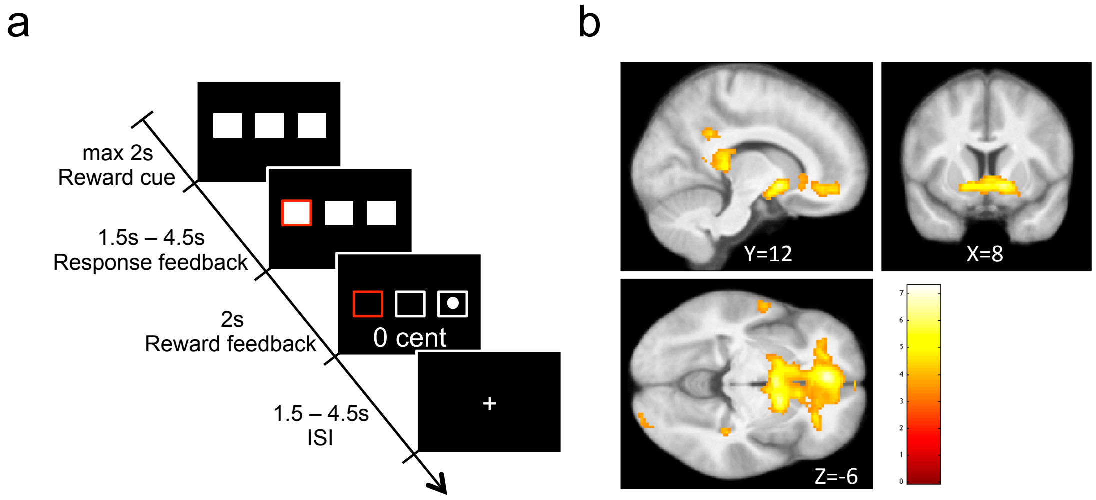

Supplementary Figure S10: Monetary decision-making task and brain responses to experienced value. (a) Screenshots show events within one trial with durations in seconds. Each trial started with the display of boxes, and participants had to make a choice within 2 seconds. The number of boxes indicated the expected reward (e.g., one chance of reward out of three). As soon as they chose one box their choice was highlighted for a jittered duration of 1.5 to 4.5 seconds. Next, the reward feedback was displayed on the screen for 2 seconds. Trials were separated by a jittered inter-trial interval (ITI) during which a fixation cross was displayed on the screen. **(b)** Statistical parametric maps (SPMs) show significant voxels in yellow and orange at time of monetary reward outcome in response to experienced value. SPMs are thresholded at $p < 0.001$ uncorrected for display and superimposed on the average anatomical brain image. MNI coordinates depict the maximum of interest in each contrast.

Supplementary Table S6: Experienced value–related activations at time of reward outcome during the monetary decision-making task

Region	BA	Size	*x*	*y*	*z*	*Z*
ACC vmPFC Ventral striatum	10	4624	–6	44	–8	5.58
	11		–2	36	–16	5.18
			12	8	–6	5.09
PCC		3665	–8	–52	14	5.05
Precuneus	39, 19, 7	359	–40	–74	44	4.51
MFG	8	253	–16	48	48	4.35
OG	18	218	30	–96	4	4.51
MTG	21	201	–58	–10	–14	4.73

Note: The table was obtained using parametric modulation by reward following outcome onset. Regions listed survived a whole-brain threshold of $p < 0.001$ uncorrected at the voxel level and $p_{FWE} < 0.05$. ACC: anterior cingulate cortex; vmPFC: ventromedial prefrontal cortex; PCC: posterior cingulate cortex; MFG: middle frontal gyrus; OG: occipital gyrus; MTG: middle temporal gyrus.

2.6. Localizing brain mediators or price cue effects on experienced taste pleasantness within brain regions of interest activated under placebo analgesia

To explore this idea we used an ROI mask that was previously reported by Wager and Atlas (2015). The ROI mask combined the vmPFC, dorsolateral prefrontal cortex, lateral orbitofrontal cortex, anterior prefrontal cortex, nucleus accumbens and ventral striatum, amygdala, hypothalamus, periaqueductal grey and rostroventral medulla. It was used to mask mediating brain regions activating in path a x b+cov(a,b). This analysis corresponds to a conjunction of the ROI mask with the mediating brain responses. Because of the exploratory purpose of this analysis and the fact that the sample size was relatively small, we applied a less conservative significance threshold of $p < 0.001$ uncorrected.

We found that brain mediators in the anterior PFC, the nucleus accumbens/ventral striatum and the vmPFC were indeed located within this ROI mask (Supplementary Table S7). This overlap of activations may suggest the existence of a common neural signature for expectancy effects across sensory domains from pain to pleasure.

Supplementary Table S7: Mediating path a x b activations within brain regions of interest underpinning increased activation under placebo analgesia.

Region	Size	*x*	*y*	*z*	*Z*
vmPFC	3	-8	44	-24	7.87
	36	-4	38	-16	7.74
Anterior PFC	4	30	64	-6	7.55
dmPFC	2	30	26	32	8.14
Ventral striatum	5	14	6	-18	7.72
	3	-8	14	-14	7.31
Premotor cortex	2	38	14	50	8.01

Note: The table was obtained using whole-brain multilevel mediation analysis at onset of wine tasting in N = 30 subjects. All regions listed survived a whole-brain threshold of $p < .001$ uncorrected.

3. Additional tables

Supplementary Table S8: Linear mixed-effects model for reaction times during fMRI scanning

Model information	Number of Observations	Fixed effects coefficients	Random effects coefficients	Covariance parameters			
	3225	12	30	2			
	AIC	BIC	Log Likelihood	Deviance			
	51337	51422	-25654	51309			
						95% CI	
Fixed effects predictors	β	SD	*t*	DF	*p*	LL	UL
Intercept	1155.7	102.34	11.3	3213	<.001	955.03	1356.33
Trial number	−98.3	12.58	−7.81	3213	<.001	−122.97	−73.63
Wine	0.76	11.95	0.06	3213	.95	−22.68	24.20
Price cue	−10.35	12.57	−0.82	3213	.41	−35.01	14.29
Payment condition	−24.83	12.57	−1.97	3213	.05	−49.49	−0.18
Trial number by Wine	17.04	12.66	1.35	3213	.18	−7.78	41.87
Trial number by Price cue	−7.35	12.56	−0.59	3213	.56	−31.97	17.27
Wine by Price cue	7.06	12.17	0.58	3213	.56	−16.79	30.92
Trial number by Payment condition	9.08	12.67	0.72	3213	.47	−15.76	33.92
Wine by Payment condition	5.38	12.51	0.43	3213	.67	−19.14	29.91
Price cue by Payment condition	11.6	12.63	0.92	3213	.36	−13.16	36.37
Trial number by Price cue by Payment condition	−10.4	12.61	−0,08	3213	.93	−25.74	23.74

Random effects covariance parameters		95% CI	
Subject (30 Levels)	β	LL	UL
Intercept	0.96	0.74	1.25
Error	β	LL	UL
Residual	1.74	1.7	1.78

Note: The table was obtained by conducting a linear mixed effects (lme) regression analysis of reaction times during wine tasting, similar to the lme analysis conducted for experienced taste pleasantness ratings.

Supplementary Table S9: Price cue–related (path a) brain activations

Region	BA	Size	*x*	*y*	*z*	*Z*
vmPFC	47	19	–54	16	–2	7.82
		9	48	22	–12	7.96
		2	–4	26	–18	7.98
Ventral striatum*		17	18	16	–2	8.10
		7	–24	8	–12	7.76
PCC	31	12	6	–22	48	7.68
	23	9	6	–56	14	7.90
dlPFC		85	14	52	40	8.62
	9	25	12	42	32	8.29
Anterior PFC	10	13	48	50	–2	8.20
IFG		55	42	30	16	7.96
		50	52	10	16	8.27
		19	50	–72	–4	8.42
	45	18	–54	26	10	8.17
	44	16	–42	8	22	7.43
MTG		88	62	–38	–6	9.29
		24	64	–18	–16	8.30
	21	17	–60	–12	–16	8.18
		5	–58	–30	–14	8.13
Hippocampus	28	22	4	–32	76	8.53
Parahippocampus		22	24	–58	–10	9.67
Parietal cortex		14	–28	–48	52	7.69
	40	12	–44	–60	42	7.90
Precuneus	7	9	6	–66	42	7.52
Occipital cortex		105	14	–76	–4	8.84
		8	–30	–92	4	7.56
Cerebellum		9	0	–48	0	7.55
		8	10	–36	–16	8.72
Thalamus		18	8	–4	12	7.91
Caudate nucleus		8	–18	14	12	8.01
Insula		14	36	–2	0	7.70
M1	4	128	50	–14	52	8.84
SMA	6	29	–8	0	62	7.93
		28	30	0	68	8.55
Premotor cortex	6	50	–44	–4	48	7.89
Postcentral gyrus	2	40	36	–42	68	8.28
	3	36	42	–34	52	7.94
	1	29	–56	–28	40	8.02
	2	21	–38	–40	62	8.74
	2	19	60	–22	28	8.00
STG	22	11	–62	–4	2	7.38
	41	8	40	–34	16	7.93
	43	6	64	–14	16	7.72
Angular gyrus	39	50	–40	–56	32	8.72

Note: The table was obtained using whole-brain multilevel mediation analysis at onset of wine tasting in N = 30 subjects. Regions listed survived a whole-brain threshold of $p < .001$ uncorrected at the voxel level and *$p_{FWE} < 0.05$. BA: Brodmann area; vmPFC: ventromedial prefrontal cortex; PCC: posterior cingulate cortex; dlPFC: dorsolateral prefrontal cortex; PFC: prefrontal cortex; IFG: inferior frontal gyrus; MTG: mid-temporal gyrus; M1: primary motor cortex; SMA: supplementary motor area; STG: superior temporal gyrus.

Supplementary Table S10: Experienced pleasantness predicting (path b) brain activations

Region	BA	Size	*x*	*y*	*z*	*Z*
Anterior PFC*	10	20	–22	62	–8	8.48
	10	6	18	64	0	7.39
ACC	24, 3	28	10	50	10	8.02
Ventral striatum*		9	16	18	–12	8.08
dlPFC	8	92	–24	18	48	8.27
	8	16	50	16	32	8.09
dmPFC	8	216	2	44	46	8.80
Hippocampus		42	–34	–32	–6	8.69
MTG	21	8	60	–18	–16	7.64
TPJ	7	6	50	–54	34	7.60
Posterior Insula	13	12	38	–10	20	7.91
Precuneus	7	24	14	–46	44	9.02
M1	4	291	40	–20	56	8.14
SMA		66	14	–18	56	8.46
		38	30	14	56	8.16
	6	32	–40	6	46	8.93
		6	48	0	32	7.46

Note: The table was obtained using whole-brain multilevel mediation analysis at onset of wine tasting in N = 30 subjects. Regions listed survived a whole-brain threshold of $p < .001$ uncorrected at the voxel level and $*p_{\mathrm{FWE}} < 0.05$. ACC: anterior cingulate cortex; dmPFC: dorsomedial prefrontal cortex; TPJ: temporal-parietal junction.

Supplementary Table S11: Brain mediators of price cue effects on experienced pleasantness ratings (i.e., path a x b)

Region	BA	Size	*x*	*y*	*z*	*Z*
vmPFC*		37	–4	38	–16	7.73
		4	6	28	–24	7.22
Ventral striatum*		7	14	8	–16	7.87
Anterior PFC*	10	9	28	64	–6	7.83
	10	4	–30	62	–4	8.27
MTG	21	9	54	0	–22	7.80
TPJ	7	5	52	–52	20	7.52
Insula		6	32	10	12	7.79
Thalamus		10	–16	–26	12	7.77
M1	4	7	40	–18	42	7.36
Occipital cortex	18	7	24	–86	–2	9.63
		6	40	–80	–2	8.04

Note: The table was obtained using whole-brain multilevel mediation analysis at onset of wine tasting in N = 30 subjects. Regions listed survived a whole-brain threshold of $p < .001$ uncorrected at the voxel level and $*p_{\mathrm{FWE}} < 0.05$ (small volume corrected).

Supplementary Table S12: Correlation of BVS reward responses and cue-related path a brain activations

Region	BA	Size	*x*	*y*	*z*	*Z*
mPFC/vmPFC*<br>vmPFC/mOFC*	10	46<br>2	6<br>–6	56<br>54	14<br>–18	8.35<br>7.79
lOFC		14<br>6	–40<br>40	28<br>32	–8<br>–10	7.53<br>8.31
Anterior PFC	10	4	26	54	14	8.00
ACC		3	0	14	22	7.23
PCC		7	–12	–50	18	8.59
Ventral striatum		6	14	4	2	8.24
dmPFC	8	13	–8	36	58	8.66
Amygdala		6<br>3	30<br>32	2<br>2	–18<br>–28	7.54<br>8.15
Insula		11	34	2	–2	8.35
MTG		21	60	–58	0	8.57
STG		7	–52	–18	–4	7.42
PAG		16<br>6	6<br>2	–30<br>–20	–12<br>–12	7.77<br>9.96

Note: The table was obtained using whole-brain multilevel, moderated mediation analysis at onset of wine tasting in a subset of N = 17 subjects. All regions listed were positively moderated in their responses to price cue by BVS responses to reward during the monetary decision-making task. They survived a whole-brain threshold of $p < .001$ uncorrected at the voxel level. *$p_{FWE} < 0.05$. mOFC: medial orbitofrontal cortex; lOFC: lateral orbitofrontal; PAG: periaqueductal grey.

Supplementary Table S13: Demographics

**Age and gender information**	
Female	15
Male	15
Age (s.e.m.) in years	29.6 (1.6)
**Professional situation**	
Student	18
Retired	0
Employed	9
Self-employed	0
Unemployed	1
Other	0
No response	2
**Annual gross income**	
< €15,000	14
€15,000 to €24,999	6
€25,000 to €34,999	0
€35,000 to €49,999	1
€50,000 to €74,999	2
€75,000 to €10,0000	0
> €10,0000	0
No response	7

Supplementary Table S14: Wine-drinking habits

	Mean	SEM
How much do you like red wine?	5.6	0.1
How much of a wine expert are you?	2.8	0.2
**How much do you agree to the following statements?**	2.0	0.03
An €18 red wine tastes better than a €3 red wine.	4.9	0.4
A German white wine tastes better than a French white wine.	5.4	0.4
Wine from a Tetra Pak tastes not as well as wine from a bottle.	3.2	0.4
Wine from a bottle with a cork tastes as good as wine from a bottle with a screw plug.	5.4	0.4
Number of glasses per week	1.9	0.2
WTP ordinary in €	6.5	0.4
WTP special occasion in €	24.8	2.7

Note: One week before the fMRI experiment, participants were asked to fill out a Qualtrics questionnaire designed to screen wine drinking habits. The aim of this procedure was to identify participants who appreciated drinking red wine but were not oenology experts. Participants rated liking of red wine and wine expertise on a seven-point Likert scale ranging from *not at all* to *a lot* for liking, and from *no expert/novice* to *expert* for wine expertise. Thus, higher scores indicate more liking for red wine and greater expertise in wine-related issues. The agreement to the statements listed in the table was given on a nine-point Likert scale (*agree* to *disagree*). Higher scores indicate greater disagreement. WTP = willingness to pay.

Supplementary Table S15: Positive and Negative Affect Schedule (PANAS) ratings and happiness ratings before and after fMRI scanning

**Pre-fMRI**	**Mean**	**SEM**	**Post-fMRI**	**Mean**	**SEM**
Positive affect	3.33*	0.09	Positive affect	3.10*	0.12
Negative affect	1.16	0.02	Negative affect	1.17	0.04
Pre-fMRI happiness	4.9	0.18	Pre-fMRI happiness	4.83	0.19

Note: Ratings on the PANAS were given on a five-point Likert scale (*not at all, a little, moderate, some, a lot*). Happiness ratings were given on a seven-point Likert scale (*not at all* to *a lot*). * Paired *t*-tests revealed a significant difference between pre- and post-fMRI scanning in positive affect ($t(29) = 3.36$, $p < .001$), with higher positive affect ratings pre-scanning compared to post-scanning. No significant differences between pre- and post-fMRI scanning were found for negative affect ($t(29) = –0.33$, $p > 0.25$) and happiness ratings ($t(29) = 0.62$, $p > 0.25$).

Supplementary Table S16: Behavioural Approach Subscale (BIS/BAS)

	Mean	SEM
BAS-DRIVE	12.7	0.3
BAS-FUN	11.8	0.4
BAS-TOTAL	27.8	3.6
BAS-REWARD	12.4	1.3

Note: Participants rated the BAS statements on a four-point Likert scale (*strong disagree, disagree, agree, strong agree*). Scores are averages of individual total sums. Individual differences on this scale did not moderate our results.

Supplementary Table S17: Tightwad-Spendthrift Scale (TW-ST)

Tightwad to Spendthrift	5.4	0.4
Spendthrift	4.3	0.5
Tightwad	2.2	0.2

Note: Participants rated their consumer behaviour between tightwad and spendthrift on an 11-point Likert scale (1–5 *tightwad*, 6 *neutral*, 7–11 *spendthrift*). In addition, spendthrift and tightwad tendencies were also rated separately on a five-point Likert scale (*never, rarely, sometimes, often, always*). Individual differences on this scale did not moderate our results.

Supplementary Table S18. fMRI data quality session 1

Sub = subject number; Vol = number of volumes; % mean ghost = percentage of the average signal outside the brain/average total signal; SNR mean = average Cohen's d (signal over noise) across time (temporal SNR) within brain; SNR std = standard deviation of SNR within brain; 95% CI = 95% confidence range for SNR within brain. x, y, z = movement directions.

Session 1						Maximal translations and rotations in mm					
Sub	Vol	% mean ghost	SNR mean	SNR std	95% CI	*x*	*y*	*z*	*x*	*y*	*z*
1	745	0.07	41.08	11.61	38.37	0.243	0.430	0.429	0.007	0.008	0.009
2	736	0.09	34.23	10.61	35.80	0.469	0.543	1.779	0.028	0.012	0.012
3	735	0.11	36.79	10.90	36.09	0.820	1.256	2.293	0.015	0.023	0.012
4	741	0.09	33.93	10.69	36.50	0.500	0.355	1.019	0.021	0.020	0.005
5	732	0.07	37.50	11.49	39.12	0.297	0.403	1.280	0.016	0.014	0.007
6	733	0.07	35.33	11.21	37.74	0.364	0.387	0.811	0.019	0.007	0.008
7	727	0.07	34.35	12.19	40.60	0.358	0.530	1.151	0.019	0.010	0.013
8	735	0.07	29.74	10.03	33.82	1.779	1.865	2.638	0.057	0.020	0.014
9	736	0.07	34.53	11.82	40.48	0.713	0.385	1.284	0.018	0.024	0.009
10	731	0.09	34.21	10.86	35.48	0.320	0.632	0.788	0.012	0.015	0.012
11	733	0.10	32.53	10.31	34.37	1.513	1.561	1.262	0.035	0.015	0.008
12	723	0.07	36.43	11.71	39.61	1.298	1.775	2.864	0.034	0.029	0.014
13	734	0.06	31.95	11.14	37.71	0.543	1.325	3.006	0.062	0.007	0.010
14	736	0.09	31.03	8.82	29.46	0.855	2.827	1.647	0.043	0.012	0.021
15	738	0.07	37.72	13.31	43.77	1.095	0.403	1.599	0.023	0.021	0.023
16	775	0.08	30.09	9.92	32.69	1.015	0.545	1.343	0.029	0.028	0.024
17	738	0.09	32.49	10.76	36.14	0.577	0.792	1.136	0.009	0.031	0.010
18	733	0.08	38.13	12.24	41.30	0.550	0.706	0.778	0.025	0.011	0.011
19	733	0.09	39.24	13.55	45.93	0.728	0.440	1.549	0.013	0.007	0.005
20	704	0.08	30.96	10.05	33.92	0.368	0.597	1.095	0.026	0.015	0.010
21	708	0.08	32.72	10.38	35.89	0.363	0.964	1.304	0.032	0.009	0.012
22	704	0.07	34.65	11.63	39.59	0.714	0.550	1.343	0.043	0.006	0.015
23	699	0.08	33.06	10.87	37.16	0.796	0.491	1.008	0.033	0.013	0.013
24	704	0.09	33.49	9.35	31.26	0.706	0.509	0.867	0.016	0.011	0.008
25	702	0.07	30.99	9.02	30.79	1.377	2.223	1.465	0.050	0.019	0.020
26	699	0.08	34.41	10.69	36.43	1.242	1.310	0.970	0.020	0.021	0.015
27	707	0.08	31.72	10.81	36.11	0.787	0.682	1.921	0.032	0.015	0.007
28	607	0.09	36.92	10.81	36.84	0.821	0.707	1.222	0.014	0.026	0.013
29	710	0.09	34.35	10.40	35.01	0.478	0.823	1.801	0.022	0.017	0.005
30	701	0.10	32.11	9.14	30.85	0.919	0.216	0.957	0.020	0.013	0.031

Supplementary Table S19. fMRI data quality session 2

Session 2						Maximal translations and rotations in mm					
Sub	Vol	% mean ghost	SNR mean	SNR std	95% CI	*x*	*y*	*z*	*x*	*y*	*z*
1	733	0.07	41.85	12.27	40.79	0.243	0.267	0.312	0.010	0.011	0.005
2	738	0.09	34.55	10.71	36.51	1.198	0.753	1.817	0.030	0.024	0.021
3	738	0.11	34.79	10.33	34.39	0.874	0.545	1.808	0.042	0.021	0.009
4	629	0.09	41.73	14.82	49.19	1.313	0.759	1.524	0.020	0.021	0.015
5	761	0.07	39.79	12.03	40.86	0.857	0.533	1.139	0.029	0.008	0.009
6	728	0.07	39.93	13.70	46.13	0.601	0.397	1.727	0.021	0.016	0.013
7	732	0.07	37.96	12.95	43.18	0.255	0.384	1.163	0.017	0.010	0.011
8	743	0.07	31.83	10.28	35.15	1.769	0.960	1.537	0.027	0.031	0.011
9	731	0.08	36.41	12.52	42.59	0.384	0.249	1.335	0.016	0.007	0.005
10	736	0.09	34.11	11.72	38.62	0.899	0.422	1.321	0.023	0.021	0.012
11	738	0.11	35.90	12.05	40.81	0.920	0.658	1.396	0.013	0.007	0.013
12	728	0.07	37.86	11.28	38.29	1.312	0.868	2.264	0.034	0.046	0.015
13	777	0.06	37.35	11.92	40.65	0.442	0.368	1.421	0.030	0.010	0.004
14	737	0.09	31.32	9.00	30.01	1.279	0.842	1.254	0.036	0.011	0.007
15	735	0.07	37.03	13.83	44.88	1.027	0.207	1.758	0.014	0.016	0.013
16	738	0.08	33.86	10.66	35.74	0.593	0.357	1.151	0.018	0.008	0.014
17	735	0.09	35.98	11.80	40.04	0.234	0.452	1.410	0.010	0.009	0.007
18	738	0.09	34.37	10.68	36.21	0.702	0.781	1.644	0.038	0.008	0.011
19	733	0.09	41.66	14.92	50.82	0.235	0.576	1.531	0.016	0.007	0.006
20	701	0.08	31.23	10.88	36.72	0.277	0.661	1.018	0.024	0.009	0.006
21	701	0.08	34.92	10.71	36.69	0.660	0.434	1.311	0.035	0.008	0.008
22	689	0.07	41.11	12.17	41.12	0.345	0.325	1.042	0.017	0.005	0.005
23	702	0.08	34.35	11.68	39.94	0.680	0.489	1.829	0.040	0.007	0.014
24	706	0.09	34.66	11.61	39.59	0.478	0.276	2.056	0.014	0.004	0.012
25	696	0.07	34.55	10.59	35.81	0.425	0.388	1.490	0.024	0.012	0.015
26	691	0.08	38.57	11.34	38.49	0.458	0.485	0.758	0.021	0.017	0.012
27	716	0.08	32.88	11.33	38.35	0.470	1.714	2.119	0.047	0.016	0.006
28	705	0.08	36.02	11.21	38.02	0.440	0.581	1.690	0.029	0.010	0.010
29	705	0.09	34.10	10.81	37.07	0.780	0.362	1.039	0.028	0.006	0.014
30	702	0.11	34.38	11.43	38.05	0.782	0.197	1.162	0.025	0.012	0.019

Supplementary Table S20. fMRI data quality session 3

Session 3						Maximal translations and rotations in mm					
Sub	Vol	% mean ghost	SNR mean	SNR std	95% CI	*x*	*y*	*z*	*x*	*y*	*z*
1	734	0.07	40.08	13.84	46.43	0.418	0.801	2.126	0.013	0.014	0.006
2	742	0.09	32.01	9.37	31.70	0.404	0.777	1.512	0.023	0.017	0.016
3	800	0.11	39.83	11.91	39.91	0.617	0.625	2.094	0.026	0.028	0.020
4	740	0.09	31.70	10.66	36.27	1.314	1.265	2.396	0.019	0.030	0.007
5	730	0.07	39.83	13.34	45.10	0.630	0.638	1.025	0.019	0.029	0.009
6	733	0.07	33.70	11.28	38.38	0.577	0.586	1.248	0.024	0.011	0.012
7	736	0.07	33.20	11.31	37.83	0.424	0.819	1.097	0.041	0.006	0.008
8	739	0.07	32.86	10.30	34.98	2.063	0.829	1.458	0.015	0.018	0.022
9	742	0.07	41.00	15.08	51.07	0.601	0.304	1.105	0.017	0.013	0.014
10	734	0.08	37.16	12.88	43.16	0.958	0.594	2.125	0.015	0.023	0.014
11	732	0.10	36.03	11.72	39.35	1.120	1.190	1.284	0.025	0.015	0.009
12	735	0.07	41.48	13.20	44.83	0.887	0.922	2.858	0.043	0.039	0.010
13	740	0.06	31.97	11.18	38.20	0.666	0.635	2.776	0.065	0.011	0.009
14	734	0.09	36.33	9.82	33.09	0.474	1.334	1.972	0.023	0.014	0.014
15	732	0.07	32.61	11.65	38.57	1.852	0.617	1.719	0.021	0.019	0.025
16	735	0.08	30.66	10.85	35.98	0.242	0.694	1.604	0.029	0.017	0.009
17	734	0.09	33.63	11.44	38.86	0.502	0.709	2.244	0.017	0.013	0.017
18	733	0.08	36.82	12.01	40.87	1.071	0.640	1.304	0.037	0.014	0.023
19	746	0.09	40.59	12.21	41.34	0.230	1.114	0.742	0.021	0.012	0.009
20	699	0.08	32.89	10.44	35.41	0.605	0.360	1.343	0.017	0.009	0.015
21	699	0.08	34.20	11.24	39.17	0.741	0.631	2.398	0.039	0.008	0.010
22	695	0.08	38.03	11.63	39.34	0.311	0.328	0.794	0.025	0.005	0.008
23	692	0.08	30.41	9.85	33.73	0.469	0.616	1.895	0.073	0.015	0.015
24	707	0.09	34.38	10.14	33.86	0.576	0.523	1.223	0.020	0.012	0.009
25	687	0.07	33.67	10.13	33.74	0.629	0.586	1.060	0.027	0.010	0.015
26	624	0.09	33.67	10.31	35.66	0.634	0.810	2.045	0.034	0.011	0.007
27	538	0.08	33.83	11.10	37.78	0.391	0.734	1.319	0.052	0.019	0.018
28	700	0.08	34.82	10.40	35.42	0.819	0.514	1.043	0.018	0.007	0.008
29	704	0.09	34.57	10.27	35.03	0.473	0.369	0.961	0.021	0.007	0.016
30	700	0.10	33.91	10.03	33.32	0.509	0.541	1.198	0.026	0.020	0.008

References

1 Bartra, O., McGuire, J. T. & Kable, J. W. The valuation system: A coordinate-based meta-analysis of BOLD fMRI experiments examining neural correlates of subjective value. *NeuroImage* **76**, 412–427 (2013).

2 Plassmann, H., O'Doherty, J., Shiv, B. & Rangel, A. Marketing actions can modulate neural representations of experienced pleasantness. *Proc. Natl. Acad. Sci. USA* **105**, 1050–1054 (2008).

3 Wager, T.D., Atlas, L.Y.. The neuroscience of placebo effects: Connecting context, learning and health. *NatRevNeuroscience* **16**(7), 404–18 (2015).
